# Phase I study of intravenously applied bispecific antibody in renal cell cancer patients receiving subcutaneous interleukin 2.

**DOI:** 10.1038/bjc.1994.366

**Published:** 1994-10

**Authors:** B. J. Kroesen, J. Buter, D. T. Sleijfer, R. A. Janssen, W. T. van der Graaf, T. H. The, L. de Leij, N. H. Mulder

**Affiliations:** University Hospital Groningen, Department of Clinical Immunology, The Netherlands.

## Abstract

In a phase I trial the toxicity and immunomodulatory effects of combined treatment with intravenous (i.v.) bispecific monoclonal antibody BIS-1 and subcutaneous (s.c.) interleukin 2 (IL-2) was studied in renal cell cancer patients. BIS-1 combines a specificity against CD3 on T lymphocytes with a specificity against a 40 kDa pancarcinoma-associated antigen, EGP-2. Patients received BIS-1 F(ab')2 fragments intravenously at doses of 1, 3 and 5 micrograms kg-1 body weight during a concomitantly given standard s.c. IL-2 treatment. For each dose, four patients were treated with a 2 h BIS-1 infusion in the second and fourth week of IL-2 therapy. Acute BIS-1 F(ab')2-related toxicity with symptoms of chills, peripheral vasoconstriction and temporary dyspnoea was observed in 2/4 and 5/5 patients at the 3 and 5 micrograms kg-1 dose level respectively. The maximum tolerated dose (MTD) of BIS-1 F(ab')2 was 5 micrograms kg-1. Elevated plasma levels of tumour necrosis factor alpha (TNF-alpha) and interferon gamma (IFN-gamma) were detected at the MTD. Flow cytometric analysis showed a dose-dependent binding of BIS-1 F(ab')2 to circulating T lymphocytes. Peripheral blood mononuclear cells (PBMCs), isolated after treatment with 3 and 5 micrograms kg-1 BIS-1, showed increased specific cytolytic capacity against EGP-2+ tumour cells as tested in an ex vivo performed assay. Maximal killing capacity of the PBMCs, as assessed by adding excess BIS-1 to the assay, was shown to be decreased after BIS-1 infusion at 5 micrograms kg-1 BIS-1 F(ab')2. A BIS-1 F(ab')2 dose-dependent disappearance of circulating mononuclear cells from the peripheral blood was observed. Within the circulating CD3+ CD8+ lymphocyte population. LFA-1 alpha-bright and HLA-DR+ T-cell numbers decreased preferentially. It is concluded that i.v. BIS-1 F(ab')2, when combined with s.c. IL-2, has a MTD of 5 micrograms kg-1. The treatment endows the T lymphocytes with a specific anti-EGP-2-directed cytotoxic potential.


Br. J. Cancer (1994), 70, 652-661 © Macmillan Press Ltd., 1994

### Phase I study of intravenously applied bispecific antibody in renal cell cancer patients receiving subcutaneous interleukin 2

B.J. Kroesen[1], J. Buter[2], D. Th. Sleijfer[2], R.A.J. Janssen[1], W.T.A. van der Graaf[2], T.H. The[1], L. de Leij[1] & N.H. Mulder[2]

University Hospital Groningen, Departments of [1]Clinical Immunology and [2]Medical Oncology, Oostersingel 59, 9713 EZ Groningen, The Netherlands.

**Summary** In a phase I trial the toxicity and immunomodulatory effects of combined treatment with intravenous (i.v.) bispecific monoclonal antibody BIS-1 and subcutaneous (s.c.) interleukin 2 (IL-2) was studied in renal cell cancer patients. BIS-1 combines a specificity against CD3 on T lymphocytes with a specificity against a 40 kDa pancarcinoma-associated antigen, EGP-2. Patients received BIS-1 F(ab')$_2$ fragments intravenously at doses of 1, 3 and 5 $\mu$g kg$^{-1}$ body weight during a concomitantly given standard s.c. IL-2 treatment. For each dose, four patients were treated with a 2 h BIS-1 infusion in the second and fourth week of IL-2 therapy. Acute BIS-1 F(ab')$_2$-related toxicity with symptoms of chills, peripheral vasoconstriction and temporary dyspnoea was observed in 2/4 and 5/5 patients at the 3 and 5 $\mu$g kg$^{-1}$ dose level respectively. The maximum tolerated dose (MTD) of BIS-1 F(ab')$_2$ was 5 $\mu$g kg$^{-1}$. Elevated plasma levels of tumour necrosis factor $\alpha$ (TNF-$\alpha$) and interferon gamma (IFN-$\gamma$) were detected at the MTD. Flow cytometric analysis showed a dose-dependent binding of BIS-1 F(ab')$_2$ to circulating T lymphocytes. Peripheral blood mononuclear cells (PBMCs), isolated after treatment with 3 and 5 $\mu$g kg$^{-1}$ BIS-1, showed increased specific cytolytic capacity against EGP-2$^+$ tumour cells as tested in an *ex vivo* performed assay. Maximal killing capacity of the PBMCs, as assessed by adding excess BIS-1 to the assay, was shown to be decreased after BIS-1 infusion at 5 $\mu$g kg$^{-1}$ BIS-1 F(ab')$_2$. A BIS-1 F(ab')$_2$ dose-dependent disappearance of circulating mononuclear cells from the peripheral blood was observed. Within the circulating CD3$^+$CD8$^-$ lymphocyte population, LFA-1$\alpha$-bright and HLA-DR$^+$ T-cell numbers decreased preferentially. It is concluded that i.v. BIS-1 F(ab')$_2$, when combined with s.c. IL-2, has a MTD of 5 $\mu$g kg$^{-1}$. The treatment endows the T lymphocytes with a specific anti-EGP-2-directed cytotoxic potential.

Interleukin 2 (IL-2)-based immunotherapy has reproducible activity in selected tumours. Renal cell carcinoma (RCC) and melanoma are the malignancies most sensitive to this form of treatment. Objective responses are observed in approximately 20% of patients with RCC, with durable complete remissions occurring in 5% (Rosenberg *et al.*, 1989). Subcutaneous (s.c.) administration of IL-2 has been found to prevent an important part of the toxicity associated with the use of intravenous (i.v.) IL-2 and has been shown to give rise to immunological and anti-tumour effects similar to the i.v. treatment (Sleijfer *et al.*, 1992; Janssen *et al.*, 1993). Still, most RCC and melanoma patients and almost all patients with other tumour types do not respond to IL-2 treatment.

A new form of immunotherapy in which the specific binding properties of monoclonal antibodies (MAbs) and the killing capacities of cytotoxic effector cells are combined, using bispecific monoclonal antibodies (BsMAbs), has been the subject of several recent studies. BsMAbs used in this treatment concept are composed of the antigen-binding subunits of two different MAbs, giving rise to one antibody with two specificities. BsMAbs which combine specificities against triggering molecules on cytotoxic effector cells on the one hand and tumour-associated antigens (TAAs) present on target cells on the other are able to redirect the lytic capcity of the effector cell towards a chosen tumour target cell (Bolhuis *et al.*, 1991). This concept has been studied *in vitro* and *in vivo* for a number of different effector and target cell populations (Kerr *et al.*, 1990; Ferrini *et al.*, 1991; Mezzanzanica *et al.*, 1991; Segal *et al.*, 1991; Weiner & Hillstrom, 1991; Weiner *et al.*, 1993). Results indicate that BsMAb-redirected effector cells can specifically kill tumour cell lines *in vitro* and established tumours *in vivo* in animal models. A start has been made to exploit this treatment modality for the treatment of cancer patients. In glioma and carcinoma patients, BsMAbs were shown to induce anti-tumour activity as well as inflammatory reactions upon local transfer of *ex vivo*-activated autologous peripheral blood mononuclear cells (PBMCs) preincubated with a BsMAb reactive with the CD3 complex on T lymphocytes and a TAA present on the tumour cells (Nitta *et al.*, 1990; Bolhuis *et al.*, 1992; Kroesen *et al.*, 1993). To investigate the therapeutic possibilities of BsMAbs further, i.e. for systemic anti-tumour treatment, we have started a phase I study of i.v. administration of BsMAb. The BsMAb used, BIS-1, is reactive with both the CD3 complex on all T lymphocytes and a pancarcinoma-associated antigen called epithelial glycoprotein (EGP-2) (De Leij *et al.*, 1993). Antibodies recognising this carcinoma-associated protein have been clustered as SCLC cluster 2 and include CO17-1A and AUA-1 (Herlyn *et al.*, 1979; Beverly *et al.*, 1988; De Leij *et al.*, 1993). EGP-2 is a membrane-bound 40 kDa glycoprotein which is highly expressed by most carcinomas and is not shed from the cell membrane (Steplewski *et al.*, 1981). EGP-2 was also found to be present on all renal cell carcinomas tested by us, although often to a low extent (our own observation). Its expression on normal tissue is restricted to simple epithelia (Varki *et al.*, 1984), and the antigen is described in a number of clinical studies as a target antigen for MAb-based immunotherapies (Sindelar *et al.*, 1986; Samonigg *et al.*, 1992; Kroesen *et al.*, 1993). In the present study BIS-1 was combined with s.c. IL-2 treatment. This combination was chosen because target cell lysis by BsMAb-redirected cytotoxic T lymphocytes (CTLs) has been shown to be dependent on preactivation of lymphocytes (Bach *et al.*, 1989; De Jong *et al.*, 1990; Kerr *et al.*, 1990). This implies that effective *in vivo* targeting of T lymphocytes towards tumour cells by BsMAb should meet the condition of prior immune activation which, in the case of local treatment settings, can be done by *ex vivo* activation of autologous immune cells (Nitta *et al.*, 1990; Weiner & Hillstrom, 1991). Immune activation *in vivo*, including T-cell activation, can be attained by s.c. IL-2 therapy (Janssen *et al.*, 1993). Furthermore, homing of lymphocytes into tumour tissue, which appears to be a prerequisite for effective cellular immunotherapy, might be enhanced by IL-2 (Fisher *et al.*, 1989; Pankonin *et al.*, 1990).

Correspondence: J. Buter, Department of Medical Oncology, University Hospital Groningen, Oostersingel 59, 7913 EZ Groningen, The Netherlands.
Received 23 February 1994; and in revised form 16 May 1994.

The present phase I trial studies the feasibility, toxicity and immunomodulatory effects of i.v.-administered BIS-1 in combination with s.c.-given IL-2 in patients with disseminated RCC who were unresponsive to previous single-agent s.c. IL-2 treatment. $F(ab')_2$ fragments of BIS-1 were made and used in the present study to prevent possible aspecific toxicity resulting from the interaction of the Fc part of the BsMAb with $FcR^+$ cells such as monocytes and the CD3-recognising part of the antibody with T cells, resulting in a cross-linking of the two cell types.

### Materials and methods

### *Patients*

All patients had a histologically confirmed diagnosis of disseminated RCC not responding to single-agent s.c. IL-2. The patients had bidimensionally measurable tumour lesions, a performance status of <2 (World Health Organization, WHO scale), an age of >18 years, an estimated life expectancy of more than 3 months, a rest period of at least 2 weeks after previous immunotherapy and an adequate haematological function (white blood count $>4,000\,mm^{-3}$, platelet count $>120,000\,mm^{-3}$, haematocrit >30%). Patients with uncontrollable disease apart from the tumour, with renal dysfunction as indicated by serum creatinine level $>120\,\mu mol\,l^{-1}$ or with hepatic dysfunction as indicated by serum bilirubin levels $>30\,mmol\,l^{-1}$ were excluded. Additional exclusion criteria were concurrent treatment with corticosteroids or prior treatment with mouse antibodies. The study was approved by the University Hospital Groningen Medical Ethical Committee. Written informed consent was obtained from all patients before the start of treatment.

### *Preparation and purification of BIS-1*

The BIS-1-producing quadroma was made in our department by fusion of the hybridomas RIV-9 and MOC-31, producing anti-CD3 (IgG3) and anti-EGP-2 (IgG1) antibodies respectively, according to a procedure described by De Lau *et al.* (1989). Large-scale production of BIS-1 was done in a hollow fibre culture system (Endotronics, Minniapolis, MN, USA) under good manufacturing practice (GMP) guidelines. Purification of the hybrid antibodies (IgG3/IgG1) from parental-type antibodies (IgG3 and IgG1), also produced by the quadroma, was done by protein A column chromatography. Hollow fibre culture supernatant was loaded onto the column at pH 7.3 and the different IgG fractions were eluted successively by lowering the pH stepwise. The BIS-1-containing fraction, eluted with 0.1 M sodium acetate, pH 4.0, was then digested by pepsin (Worthington, Freehold, NJ, USA) using a final BIS-1-pepsin ratio of 100:1 (w/w). Digestion was performed at 37°C for 4 h, immediately followed by G150 Sephadex gel filtration to separate BIS-1 $(F(ab')_2$ from undigested IgG, fragmented Fc portions and pepsin. The purified BIS-1 $F(ab')_2$ solution was adjusted to a concentration of $0.2\,mg\,ml^{-1}$ with 0.9% sodium chloride. HSA (Institute Merieux, Lyon, France) was added to a concentration of 0.5% and the preparation was then passed through a 0.22 μm filter and stored sterile at 4°C. Sterility of the BIS-1 $F(ab')_2$ preparation was confirmed by culturing in Clausur medium. The BIS-1 $F(ab')_2$ preparation was pyrogen free as tested in *Limulus* amoebocyte lysate assay and by intravenous administration of the preparation to rabbits. Abnormal toxicity was tested for by administration of the BIS-1 $F(ab')_2$ preparation to mice and guinea pigs both intravenously and intraperitoneally according to the protocol of the Dutch Pharmacopee IX (1980) and was found to be absent. The ability of the BIS-1 $F(ab')_2$ preparation to redirect the lytic activity of T lymphocytes towards EGP-2-positive tumour cells was assessed in a standard $^{51}Cr$-release assay in which *in vitro*-activated T lymphocytes (effector lymphocytes, see below) were used as effector cells and GLC-1M13 (EGP-2-positive), GLC-1 (EGP-2-negative) and P815 (FcR-positive) were used as target cells. The target cell line P815 was used to check whether or not the BIS-1 $F(ab')_2$ preparation was devoid of undigested Fc containing BIS-1 IgG.

### *Treatment*

Patients received daily subcutaneous injections of $18 \times 10^6$ IU of IL-2 (Proleukin, EuroCetus Amsterdam, The Netherlands) in a 5 day weekly cycle for four consecutive weeks as previously described (Sleijfer *et al.*, 1992). The dose in the first 2 days of the second, third and fourth weeks was reduced to $9 \times 10^6\,IU\,day^{-1}$ followed by 3 days of $18 \times 10^6\,IU\,day^{-1}$. Acetaminophen 250–500 mg orally every 4–6 h was given to suppress pyretic reactions. The BIS-1 $F(ab')_2$ BsMAb was administered in 100 ml of 0.9% sodium chloride as a 2 h i.v. infusion.

### *Study protocol*

Consecutive cohorts of at least four patients were treated at each dose level in a dose-escalating phase I trial design. Dose levels of 1, 3 and $6\,\mu g\,kg^{-1}$ body weight were planned to be studied. The antibody was administered in the rebound phase of a preceding 5 day s.c. IL-2 cycle when the number of peripheral blood T lymphocytes was high (Janssen *et al.*, 1993). The first two patients from a particular dose level received the antibody on days 8 and 22 and the following two patients received the antibody on days 8 and 23, in order to study the effects of the antibody both before (day 8 and 22) and during (day 23) a cycle of IL-2 administration. On the day of the antibody infusion, IL-2 was administered 4 h after the end of infusion. The end point of the study was dose-limiting toxicity, defined as toxicity exceeding WHO grade II. The maximum tolerated dose (MTD) was defined as the dose level below that producing dose-limiting toxicity. Immunological parameters, including cytological and functional binding of BIS-1 $F(ab')_2$, were monitored before and at the end of the antibody infusion. Quantification of BIS-1 $F(ab')_2$ binding to T lymphocytes was done by a flow cytometry-based procedure. Functional binding of BIS-1 $F(ab')_2$ to T lymphocytes was assessed in a standard $^{51}Cr$-release cytotoxicity assay against the target cell lines GLC-1M13 (EGP-2-positive) and GLC-1 (EGP-2-negative).

### *Toxicity and response monitoring*

Before treatment, patients were staged with a full physical examination, determination of WHO performance status, renal, liver, thyroid and haematological function, an ECG and radiological recording of disease extension. During treatment weight and temperature were recorded daily and renal, liver and haematological functions were determined weekly. Thyroid function was determined before and after the whole treatment. Blood samples were taken before the start and at the end of infusion of BIS-1 $F(ab')_2$ for analysing BIS-1 $F(ab')_2$ binding to T lymphocytes and the cytotoxic capacity of isolated PBMCs *in vitro*. Vital functions were measured every 30 min during the infusion, every 2 h thereafter and three times daily in the 5 days during which the patients were hospitalised. Toxicity was scored according to standard WHO criteria (Anonymous, 1979). Dose modifications were planned as follows. In case of toxicity during the BIS-1 $F(ab')_2$ infusion exceeding WHO grade II the infusion was abrogated. In the case of weight gain >5% or an increase in serum creatinine level of more than 100% or a decrease in systolic tension of 25% during the rest of treatment, the IL-2 was discontinued. Treatment could be reinstituted when toxicity had returned to below a grade I level. If IL-2 had to be withheld for more than 5 days the patient went off study. Response was monitored by radiographic techniques as appropriate. Tumour elevations were repeated after 4 weeks of treatment and every 3 months thereafter. A complete response was defined as the disappearance of all evidence of tumour for a minimum of 4 weeks; a partial response was recorded when a 50% or greater decrease in the sum of the products of all diameters of evaluable lesions was reached. Patients with a response less than partial or an increase of less than 25% for at least 3 months were classified as having stable disease. Progression was defined as an increase of more than 25% or the development of new lesions.

*PBMC isolation*

PBMCs were obtained from heparinised peripheral blood. Isolation was done by density centrifugation of diluted (1:1 in phosphate-buffered saline PBS) blood on Lymphoprep (Nycomed, Oslo, Norway) at 2,400 r.p.m. for 20 min. The PBMC fraction was washed twice by resuspension in RPMI-1640 (Gibco Europe, Breda, The Netherlands) and centrifugation at 1,800 (first time) and 1,200 (second time) r.p.m. for 10 min. After isolation, PBMCs were collected in complete medium consisting of RPMI-1640 supplemented with 2% heat-inactivated human pooled serum, 2 mM glutamine and 60 $\mu g\ ml^{-1}$ gentamicin.

*Antibodies used for flow cytometry*

For phenotyping of T lymphocytes and assessment of BIS-1 $F(ab')_2$ binding to T lymphocytes, the following MAbs were used: fluorescein isothiocyanate (FITC)- or phycoerythrin (PE)-labelled anti-Leu-4 (CD3), biotinylated anti-Leu-2a (CD8), FITC-labelled anti-LFA-1α (CD11a), PE-labelled HLA-DR (Becton Dickinson, Mountain View, CA, USA), bitonylated goat anti-mouse Ig (GαM-biotin) and biotinylated goat anti-mouse Ig (GαM-biotin) (Southern Biotechnologies, Cambridge, MA, USA).

*Flow cytometric analysis*

CD3 occupancy by BIS-1 $F(ab')_2$ was assessed using an indirect immunofluorescence staining procedure in which streptavidin–PE (SAPE) (Becton Dickinson) was used to amplify the BIS-1 $F(ab')_2$ detection with biotinylated goat anti-mouse antibodies (Zola *et al.*, 1990). A 100 μl aliquot of peripheral EDTA blood or isolated PBMC samples were incubated with phosphate-buffered saline (PBS) or a saturating amount of BIS-1 $F(ab')_2$ (2 $\mu g\ ml^{-1}$) at 4°C for 30 min. After one wash with 2 ml of PBS and 50 μl GαM-biotin or, as a control for aspecific binding of the conjugate, GαR-biotin (each 40 × diluted in PBS containing 1% pooled human serum) was added to the cell pellet and incubated at 4°C for 30 min. After one wash with 2 ml of PBS, 10 μl streptavidin–PE was added to the cell pellet and the samples were incubated at 4°C for 30 min. Cells were resuspended in 2 ml of FACS lysing solution (Becton Dickinson), incubated for 10 min at room temperature, washed once with PBS and resuspended in a final volume of 150 μl PBS for flow cytometric analysis. The CD3 occupancy was calculated according to the following formula:

$$\frac{\mathrm{MFI}t = x\ (\mathrm{PBS/G\alpha M\text{-}bio/SAPE}) - \mathrm{MFI}t = x\ (\mathrm{PBS/G\alpha R\text{-}bio/SAPE})}{\mathrm{MFI}t = x\ (\mathrm{BIS\text{-}1/G\alpha M\text{-}bio/SAPE}) - \mathrm{MFI}t = x\ (\mathrm{BIS\text{-}1/G\alpha R\text{-}bio/SAPE})} \times 100\%$$

in which the sequential incubation steps are given between brackets, MFI is the mean fluorescence intensity, SAPE is streptavidin–PE and $t = x$ represents either $t = 0$ h (before the infusion) or $t = 2$ h (after the infusion).

Changes in leucocyte numbers as induced by the treatment were analysed by a Coulter Leucocounter (Coulter Electronics, Hilaleah, FL, USA). Changes occurring within the CD3/CD8 double-positive cell population as induced by the treatment were analysed by three-colour flow cytometry. CD8-biotin/streptavidin–allophycocyanin (APC) and CD3-PE or CD3-FITC was used to select for CD3/CD8 double-positive T lymphocytes, and CD11a-FITC and HLA-DR-PE conjugates were used to analyse the presence of LFA-1α- and HLA-DR-positive cells within the CD3/CD8 cell population. Staining was performed on 100 μl of peripheral EDTA blood obtained from the patient just prior to ($t = 0$ h) and directly after ($t = 2$ h) BIS-1 infusion. In the first step, CD8–biotin was allowed to bind at 4°C for 30 min followed by one wash with 2 ml of PBS. The second step included the addition of either streptavidin–APC, CD3–FITC, HLA-DR–PE or streptavidin–APC, CD3–PE, CD11a-FITC to the resuspended cell pellet and incubation for another 30 min at 4°C. The cell suspension was resuspended in 2 ml of FACS lysing solution, incubated for 10 min at room temperature, washed once with 2 ml of PBS and resuspended in a final volume of 150 μl of PBS for analysis. The samples were analysed on a Coulter Elite Cytometer (Coulter Electronics) using an argon laser (488 nm) for FITC and PE excitation and a He/Ne (623 nm) laser for excitation of APC. Immunofluorescence emission was measured using a 525 nm bandpass filter for FITC, a 575 nm bandpass filter for PE and a 675 nm bandpass filter for APC.

*Effector lymphocytes*

In order to test the activity of the BIS-1 $F(ab')_2$ preparation, PBMCs isolated from healthy volunteers were activated by incubating the cells for 3 days in complete medium supplemented with 5% (giving about 0.5 $\mu g\ ml^{-1}$ IgG end concentration) culture supernatant of the mitogenic anti-CD3 MAb WT-32 (Tax *et al.*, 1983), followed by washing and incubation for two additional days in complete medium supplemented with 60 IU $ml^{-1}$ IL-2 (EuroCetus, Amsterdam, The Netherlands). To assess functional binding of BIS-1 $F(ab')_2$ to T lymphocytes in the clinical study, freshly isolated PBMCs drawn from the patients just before and after the 2 h antibody infusion were used directly in the $^{51}Cr$-release cytotoxicity assay.

*Target cell lines*

GLC-1M13 (EGP-2-positive) and GLC-1 (EGP-2-negative) are small-cell lung cancer (SCLC)-derived cell lines (De Leij *et al.*, 1985). P815 is a FcR-positive mouse mastocytoma cell line. These cell lines were cultured according to routine procedures in RPMI-1640-based medium supplemented with 14% heat-inactivated fetal calf serum, 2 mM glutamine 60 $\mu g\ ml^{-1}$ gentamicin (Schering, Kenilworth, USA), 0.05 mM β-mercaptoethanol and 1 mM sodium pyruvate at 37°C in a humidified atmosphere containing 5% carbon dioxide.

*$^{51}Cr$-release assay*

$^{51}Cr$-release assays were performed according to standard procedures to assess BIS-1-redirected T-cell cytotoxicity. All determinations were done in triplicate in the presence of 60 IU $ml^{-1}$ IL-2. Before the assay, $5 \times 10^6$ target cells (GLC-1M13, GLC-1 or P815) were suspended in 100 μl of culture medium containing 3.7 MBq of [$^{51}Cr$]sodium chromate (Amersham, UK) and incubated for 1 h at 37°C in a humidified, 5% carbon dioxide-containing atmosphere. Unbound [$^{51}Cr$]sodium chromate was removed by washing the cells three times with medium. A 50 μl aliquot of medium containing 0.4 $\mu g\ ml^{-1}$ BIS-1 $F(ab')_2$ (giving a final concentration of 0.1 $\mu g\ ml^{-1}$ during the assay) or not was pipetted into a 96-well round-bottom microtitre plate (Greiner no. 650180) and incubated with 50 μl of $2.5 \times 10^4$ or $2.5 \times 10^5$ effector lymphocytes for 15 min at room temperature. Subsequently, 100 μl of medium containing $2.5 \times 10^3$ $^{51}Cr$-labelled target cells was added to each well to give effector to target (E/T) ratios of 1, 10 and 100 in a final volume of 200 μl. The microtitre plates were centrifuged at 500 r.p.m. for 2 min and incubated at 37°C in 5% carbon dioxide for 4 h. After the incubation, the plates were centrifuged at 1,000 r.p.m. for 5 min and 100 μl samples taken from the supernatant were counted in a gamma counter for 5 min. Cell lysis was calculated from the percentage $^{51}Cr$ released, according to the formula:

$$\frac{\text{Experimental release} - \text{spontaneous release}}{\text{Maximal release} - \text{spontaneous release}} \times 100\%$$

Maximal release was determined from a sample to which 100 μl of 2% Triton X-100 solution was added instead of effector cells. Spontaneous release was determined from a sample to which 50 μl of medium was added instead of effector cells.

*Cytokine release*

Antibody-based capture ELISAs were used to assess TNF-α (British Bio-technology, Oxford, UK) and IFN-γ (Eurogenetics, Leuven, Belgium) according to the manufacturer's instructions. Blood plasma was isolated by centrifugation of EDTA-containing peripheral blood samples at 2,500 r.p.m. at 4°C for 5 min immediately after collection. The cell fraction was discarded and the plasma samples stored at −20°C until use.

*Statistics*

Changes in leucocyte count, phenotype and function were statistically analysed using the Wilcoxon test for paired observations. A two-sided α-level of 0.05 was considered significant.

### Results

In vitro *effectiveness and characteristics of BIS-1 F(ab')₂*

The purified BIS-1 F(ab')$_2$ fragment preparation used in this study was analysed by SDS-PAGE. No undigested IgG could be detected (data not shown). In agreement with this, the preparation induced no Fc-mediated cytotoxicity of activated cytotoxic T lymphocytes towards the FcR-positive target cell line P815, whereas undigested BIS-1 IgG did (Figure 1). BIS-1 F(ab')$_2$ proved to be equally as able as undigested BIS-1 IgG of redirecting cytotoxic T lymphocytes towards EGP-2-positive, but not EGP-2-negative, tumour cells. Optimal target cell lysis is induced in both cases at a concentration of 0.1 μg ml$^{-1}$ BIS-1 in which the actual BIS-1 F(ab')$_2$ concentration was corrected for its lower molecular weight. The degree to which CD3 molecules on T lymphocytes are occupied with BIS-1 at this concentration is 27%, but even at a CD3 occupancy as low as 2% (1 ng ml$^{-1}$) considerable target cell-directed cytotoxicity is induced (Figure 1).

*Phase I study*

From October 1992 until June 1993, 14 patients with advanced RCC who showed no response after 4 weeks of s.c. IL-2 treatment were entered. The characteristics of the patients are listed in Table I. All patients were eligible for evaluation of toxicity. Side-effects arising from the BIS-1 F(ab')$_2$ administration are listed in Table II. This dose-dependent toxicity could be discriminated from the toxicity associated with IL-2 by its acute and rapidly transient characteristics. At the 1 μg kg$^{-1}$ dose level no toxicity was observed. Toxicity occurred in two out of four patients at the 3 μg kg$^{-1}$ dose level and consisted of chills, peripheral vasoconstriction with rise in diastolic tension, temporary dyspnoea and fever. Symptoms started suddenly in all patients, approximately 100 min after the start of infusion, and lasted 15–30 min, except for the fever, which started approximately 30 min after the end of the infusion and lasted for 1–2 h. No neurotoxicity was observed after administration of the BIS-1 F(ab')$_2$. In the first patient at the 6 μg kg$^{-1}$ dose level, severe rigors started 1 h after the start of infusion, and became accompanied by increasing dyspnoea with signs of cyanosis, whereupon the infusion had to be abrogated. Symptoms resolved gradually over the next 4–6 h except for fever. The patient had received a total dose just over 5 μg kg$^{-1}$. On day 22, a dose of 5 μg kg$^{-1}$ could be administered to the same patient as a 2 h infusion, producing toxicity with chills to a grade I toxicity. No further dose escalation was attempted. A total of five other patients were treated at a 5 μg kg$^{-1}$ dose level, producing grade I–II toxicity in all patients. This dose was considered the MTD. No difference in toxicity was observed between the administration of the antibody on days 8 and 22, before the start of a new IL-2 cycle, or on day 23, during an IL-2 cycle. There was no correlation between sex, age, performance status, prior nephrectomy or the presence of lung metastases and toxicity.

Toxicity during subcutaneous IL-2 treatment was similar to previous findings with s.c. IL-2 monotherapy (Sleijfer *et al.*, 1992) and consisted of transient inflammation and local induration at the injection sites in all patients. The residual nodular lesions resembling subcutaneous lipomas disappeared slowly during a 2–4 month period. Flu-like symptoms with fever and chills occurred in all patients, leading to a grade I–II toxicity in 21/56 and 29/56 treatment weeks respectively. Nausea/vomiting grade I, II and III occurred during 25/56, 20/56 and 1/56 weeks respectively, while diarrhoea was observed during 5/56 weeks reaching grade I only. Hypotension was not observed. Transient elevations of γ-glutamyltranspeptidase and alkaline phosphatase were observed in 34% and 21% of patients respectively. One patient receiving

**Figure 1** Cytolytic activity of *in vitro*-activated T lymphocytes of healthy volunteers against the EGP-2-positive target cell line GLC-1M13 (●, +) and the FcR-positive target cell line P815 (▼, ▲) induced by various concentrations of BIS-1 IgG (●, ▼) and BIS-1 F(ab')$_2$ (+, ▲). Cell lysis was assessed in a 4 h $^{51}$Cr-release assay at an E:T ratio of 100:1. CD3 occupancy (×) of T lymphocytes by the indicated concentrations of BIS-1 F(ab')$_2$ was measured as described in the Materials and methods section.

**Table I** Patients characteristics

| | *n* | % |
|---|---|---|
| Patients | 14 | |
| Female/male | 6/8 | |
| Age (years) | | |
| Median | 58 | |
| Range | 48–70 | |
| WHO performance | | |
| 0 | 3 | 21 |
| 1 | 7 | 50 |
| 2 | 4 | 29 |
| Prior therapy | | |
| Nephrectomy | 6 | 43 |
| Chemotherapy | 0 | 0 |
| Immunotherapy | 14 | 100 |
| Tumour localisations | | |
| Primary | 8 | 57 |
| Lung | 9 | 64 |
| Bone | 5 | 36 |
| Other | 4 | 29 |
| Number of metastatic sites | | |
| 1 | 2 | 14 |
| 2 | 8 | 57 |
| 3 or more | 4 | 11 |

**Table II** Toxicity and response of treatment

| No. | Sex | Dose ($\mu g\ kg^{-1}$) | Toxicity 1 day 8 | Toxicity 2 day 22 or 23 | Response |
|---|---|---|---|---|---|
| 1 | M | 1 | – | – | SD |
| 2 | M | 1 | – | – | PD |
| 3 | F | 1 | – | – | SD |
| 4 | F | 1 | – | – | PD |
| 5 | F | 3 | – | – | PD |
| 6 | M | 3 | Chills, fever | Chills | PD |
| 7 | F | 3 | – | – | SD |
| 8 | M | 3 | – | Chills | PR, 6 months |
| 9 | M | 6→5[a] | Chills, dyspnoea, fever | Chills | PD |
| 10 | F | 5 | Chills, temporary, dyspnoea, fever | Chills | SD |
| 11 | M | 5 | Chills | – | PD |
| 12 | F | 5 | Chills | Chills | PD |
| 13 | M | 5 | Chills, temporary dyspnoea, fever | Chills, temporary dyspnoea, fever | PD |
| 14 | M | 5 | – | Chills, temporary dyspnoea, fever | SD |

[a]At day 8 infusion was abrogated at $t = 1.5$ h, giving an effective dose of approximately $5\ \mu g\ kg^{-1}$. At day 22 $5\ \mu g\ kg^{-1}$ was given in a 2 h infusion. F, female; M, male; PR, partial remission; SD, stable disease; PD, progressive disease.

BIS-1 (at the $5\ \mu g\ kg^{-1}$ dose level) had gradual progression of pre-existent renal dysfunction at the end of IL-2 therapy, resulting in a creatinine elevation to grade III toxicity. Thyroid dysfunction was observed in one patient. Peripheral blood lymphocyte counts dropped temporarily upon administration of the BIS-1 $F(ab')_2$, as will be discussed below, but these numbers returned to normal values 24 h after the infusion. During subsequent IL-2 treatment mean peripheral blood lymphocyte count rose from 1.8 (s.d. 1.0) to a maximum of 4.8 (s.d. 2.6) $\times 10^9\ l^{-1}$, while eosinophil counts rose from 0.3 (s.d. 0.3) to 4.9 (s.d. 3.9) $\times 10^9\ l^{-1}$. No additional toxicity, especially no enhanced mucositis, diarrhoea or skin toxicity, from the BIS-1 $F(ab')_2$ antibody on top of the IL-2-related toxicity was observed during the rest of the IL-2 treatment course when compared with previous s.c. IL-2 monotherapy (Sleijfer et al., 1992).

### *Response to treatment*

All 14 patients were assessable for response after 4 weeks of treatment. One partial response in a patient with lung metastasis who received $3\ \mu g\ kg^{-1}$ was observed and lasted 6 months. Five patients showed stable disease for at least 3 months and eight patients exhibited progressive disease.

### In vivo *occupancy of CD3 by BIS-1*

Immediately after ending the i.v. BIS-1 infusion, the degree to which available CD3 molecules on T lymphocytes were occupied with BIS-1 was assessed by flow cytometry. Figure 2 summarises the results obtained from patients treated with different doses of BIS-1 $F(ab')_2$. At $1\ \mu g\ kg^{-1}$ BIS-1 $F(ab')_2$, the presence of mouse antibodies bound to T lymphocytes was just detectable and the degree to which CD3 molecules were occupied was determined to be 1.5% (s.d. 0.4). At $3\ \mu g\ kg^{-1}$ BIS-1 $F(ab')_2$, binding of the BsMAb to T lymphocytes was clearly detectable and CD3 occupancy was 4% (s.d. 0.9), whereas at $5\ \mu g\ kg^{-1}$ BIS-1 $F(ab')_2$ CD3 occupancy was 6% (s.d. 3). Within the dose levels described no differences in CD3 occupancy were observed between the first (day 8) and the second (day 22 or 23) infusion of BIS-1 $F(ab')_2$. After PBMC isolation, in order to perform $^{51}Cr$-release assays, the CD3 occupancy by BIS-1 $F(ab')_2$ was measured again and proved to be reduced from 4% to 2.5% (s.d. 0.6) and from 6% to 3% (s.d. 0.5) for doses of 3 and $5\ \mu g\ kg^{-1}$ BIS-1 respectively. The CD3 occupancy by BIS-1 $F(ab')_2$ was found to decrease in the next hours (not shown).

**Figure 2** Mean CD3 occupancy of T lymphocytes *in vivo*, measured directly after infusion with 1 (four patients, $n = 8$), 3 (four patients, $n = 8$) and 5 (six patients, $n = 12$) $\mu g\ kg^{-1}$ BIS-1 $F(ab')_2$ (solid bars). After PBMC isolation, done to perform cytotoxicity assays, the CD3 occupancy of T lymphocytes by BIS-1 $F(ab')_2$ was measured again (□). ND, not done.

### *Functional analysis of BIS-1 binding*

The ability of *in vivo* BIS-1 $F(ab')_2$-loaded T lymphocytes to exert specific anti-tumour activity was assessed in an *in vitro* $^{51}Cr$-release assay. PBMCs isolated from peripheral blood obtained just before ($t = 0$ h) and directly after ($t = 2$ h) infusion with BIS-1 $F(ab')_2$ were incubated with $^{51}Cr$-labelled EGP-2-positive and -negative target cells at E/T ratios of 1, 10 and 100. Both at $t = 0$ h and at $t = 2$ h, the assay was also performed in the presence of additional BIS-1 $F(ab')_2$ ($0.1\ \mu g\ ml^{-1}$) added to the assay to assess the maximal redirected cytotoxic capacity of the PBMC. Figure 3 shows the results of the EGP-2-redirected cytolytic capacity of these freshly isolated PBMCs at an E/T ratio of 100. PBMCs isolated after infusion of $3\ \mu g\ kg^{-1}$ BIS-1 $F(ab')_2$ showed significantly higher specific anti-tumour activity than PBMCs isolated before the infusion ($P < 0.04$), indicating that the *in vivo* bound BIS-1 endowed the T lymphocytes with a functional anti-EGP-2 redirected cytotoxic capacity (see Figure 3a, $t = 0-$ and $t = 2-$). At this dose, maximal redirected cytotoxic capacity, before and after the infusion, as assessed by adding 0.1 $\mu g\ ml^{-1}$ BIS-1 $F(ab')_2$ *in vitro* to the assay, was approximately the same in all patients tested (see Figure 3a, $t = 0+$ and $t = 2+$). At 5 $\mu g\ kg^{-1}$, however, the maximal redirected cytotoxic capacity after the 2 h infusion was found to be significantly lower ($P < 0.005$) than the maximal redirected cytotoxic capacity before infusion using the same E/T ratio (see Figure 3b, $t = 0+$ and $t = 2+$). Using the EGP-2-negative target cell line GLC-1, no cytotoxicity could be measured at any of the assessed doses or time points, indicating that possible LAK activity did not interfere with this assay (data not shown).

**Figure 3** Percentage target cell lysis (GLC-1M13) induced by PBMCs isolated just before ($t = 0$ h) and directly after ($t = 2$ h) infusion with 3 (four patients, $n = 8$) (**a**) and 5 (six patients, $n = 12$) (**b**) $\mu g\ kg^{-1}$ BIS-1 $F(ab')_2$. Cytolytic activity was assessed in the absence ($t = 0-$) and $t = 2-$) and presence ($t = 0+$ and $t = 2+$) of *in vitro*-added 0.1 $\mu g\ ml^{-1}$ BIS-1 $F(ab')_2$. The data shown are obtained at an E/T ratio of 100:1. Median as well as 25 and 75 percentiles are shown as a box plot, whereas whiskers range down to 5 and up to 95 percentile values. Asterisk in **a** indicates a significant increase compared with the values at $t = 0-$, ($P < 0.04$, Wilcoxon test). Asterisk in **b** indicates a significant decrease compared with the values at $t = 0+$ ($P < 0.005$ Wilcoxon test).

*Treatment-related cytokine release*

Since the observed toxicity in the patients treated at the 5 $\mu g\ kg^{-1}$ BIS-1 $F(ab')_2$ dose level (Table II) might be explained by the release of secondary cytokines, serum levels of TNF-α were assessed in patients treated at 3 and 5 $\mu g\ kg^{-1}$ before and at different time points after the infusion (Figure 4). Also IFN-γ, a cytokine thought to be produced more selectively by activated T cells, was measured in these patients. At 3 $\mu g\ kg^{-1}$ no elevation of serum TNF-α or IFN-γ was found in two patients (nos. 7 and 8) tested. Administration of 5 $\mu g\ kg^{-1}$ body weight BIS-1 $F(ab')_2$, however, evoked TNF-α production up to 180 $pg\ ml^{-1}$, and IFN-γ production up to 12 $U\ ml^{-1}$ (patients 9 and 10 tested). Peak levels of TNF-α were found 2 h after the start of the infusion. Peak levels of IFN-γ were detected 3 h after the start of the infusion. Both TNF-α and IFN-γ had returned to almost base levels 24 h after the infusion.

*Leucocyte numbers and immunophenotyping*

Infusion of BIS-1 proved to have profound effects on the number of leucocytes present in the blood. Figures 5 and 6 show absolute numbers of peripheral monocytes and lymphocytes before and after the 2 h infusion with BIS-1 $F(ab')_2$. No consistent changes in the numbers of granulocytes were found to occur. At 1 $\mu g\ kg^{-1}$ BIS-1 $F(ab')_2$ no decrease in peripheral monocyte and lymphocyte numbers was observed. At 3 and 5 $\mu g\ kg^{-1}$ BIS-1 $F(ab')_2$, however, especially monocyte but also lymphocyte numbers were significantly reduced in the blood ($P < 0.01$ at 3 $\mu g\ kg^{-1}$ and $P < 0.005$ at 5 $\mu g\ kg^{-1}$). To analyse this phenomenon more specifically, blood samples obtained from patients 11–14 were stained with CD3, CD8, CD11a (LFA-1α) and anti-HLA-DR before and after infusion with 5 $\mu g\ kg^{-1}$ body weight BIS-1 $F(ab')_2$. Changes occurring within the CD3/CD8 double-positive T-lymphocyte population between the blood samples taken before and after the infusion were analysed. Within the CD3/CD8 double-positive T-cell population, LFA-1α-bright and HLA-DR-positive cells disappeared from the blood to a greater extent ($P < 0.02$ and $P < 0.01$ respectively) as a result of BIS-1 $F(ab')_2$ infusion than the LFA-1α-dim or HLA-DR-negative cells (Table III).

**Figure 4** TNF-α (■) and IFN-γ (□) levels in plasma (patient 10) after i.v. infusion with 5 $\mu g\ kg^{-1}$ BIS-1 $F(ab')_2$. The infusion was applied between $t = 0$ and $t = 2$ h.

**Figure 5** Absolute numbers of monocytes ($\times 10^6$ ml$^{-1}$) just before ($t = 0$ h) and directly after ($t = 2$ h) infusion with 1 (four patients, $n = 8$) (**a**), 3 (four patients, $n = 8$) (**b**) and 5 (six patients, $n = 12$) (**c**) μg kg$^{-1}$ BIS-1 F(ab')$_2$. Median as well as 25 and 75 percentiles are shown as a box plot whereas whiskers range down to 5 and up to 95 percentile values. Asterisk indicates a significant reduction ($P < 0.005$, Wilcoxon test) compared with the values at $t = 0$ h).

**Figure 6** Absolute numbers of lymphocytes ($\times 10^6$ ml$^{-1}$) just before ($t = 0$ h) and directly after ($t = 2$ h) infusion with 1 (four patients, $n = 8$) (**a**), 3 (four patients, $n = 8$) (**b**) and 5 (six patients, $n = 12$) (**c**) μg kg$^{-1}$ BIS-1 F(ab')$_2$. Median as well as 25 and 75 percentiles are shown as a box plot whereas whiskers range down to 5 and up to 95 percentile values. Asterisk indicates a significant reduction ($P < 0.005$, Wilcoxon test) compared with the values at $t = 0$ h).

**Table III** Immunophenotyping of peripheral blood lymphocytes before and after BIS-1 F(ab')$_2$ infusion at 5 μg kg$^{-1}$ body weight

| Patient no. | Day of treatment | | CD3[a] | CD8[b] | CD11a[c] Bright | CD11a[c] Dim | Ratio | HLA-DR[c] |
|---|---|---|---|---|---|---|---|---|
| 11 | Day 8 | $t = 0$ h | 66 | 21 | 63 | 35 | 1.8 | 49 |
| | | $t = 2$ h | 62 | 18 | 50 | 49 | 1.0 | 39 |
| | Day 23 | $t = 0$ h | 56 | 19 | 72 | 28 | 2.6 | 67 |
| | | $t = 2$ h | 58 | 17 | 44 | 36 | 1.2 | 52 |
| 12 | Day 8 | $t = 0$ h | 52 | 15 | 58 | 42 | 1.4 | 28 |
| | | $t = 2$ h | 53 | 13 | 43 | 56 | 0.8 | 12 |
| | Day 23 | $t = 0$ h | 33 | 19 | 56 | 44 | 1.3 | 12 |
| | | $t = 2$ h | 31 | 15 | 37 | 63 | 0.6 | 8 |
| 13 | Day 8 | $t = 0$ h | 58 | 26 | 68 | 32 | 2.1 | 16 |
| | | $t = 2$ h | 54 | 28 | 68 | 32 | 2.1 | 10 |
| | Day 23 | $t = 0$ h | 62 | 16 | 64 | 36 | 1.8 | 10 |
| | | $t = 2$ h | 73 | 16 | 45 | 54 | 0.8 | 4 |
| 14 | Day 8 | $t = 0$ h | 76 | 15 | 85 | 14 | 6.1 | 39 |
| | | $t = 2$ h | 83 | 14 | 67 | 32 | 2.1 | 26 |
| | Day 23 | $t = 0$ h | 64 | 21 | 76 | 24 | 3.6 | 31 |
| | | $t = 2$ h | 59 | 22 | 56 | 43 | 1.6 | 19 |

[a]Percent positive cells of total lymphocytes. [b]Percent positive cells of CD3-positive lymphocytes. [c]Percent positive cells of CD3 CD8 double-positive lymphocytes.

### Discussion

The mechanisms of *in vivo* IL-2-mediated anti-tumour responses are still not clarified. However, it is conceivable that to attain effective cellular immunotherapy a number of prerequisites should be met. These include (1) effector cell activation, (2) presence or migration of effector cells in or to the tumour site and (3) specific recognition and killing of the tumour target cells by the effector cells. During IL-2 treatment, NK effector cells as well as T lymphocytes become activated (Janssen *et al.*, 1993), and the latter cells have been shown to migrate to the site of the tumour (Fisher *et al.*, 1989; Pankonin *et al.*, 1990). However, in RCC patients, who are in fact the best responding patient group, an overall response rate of only approximately 20% to IL-2 therapy is attained (Rosenberg *et al.*, 1989). Resistance to IL-2 therapy might be due to an inability of the effector cells to specifically recognise and/or kill the tumour cells. BsMAbs that are able to bind to both tumour cells and effector cells might add such specificity to the IL-2 treatment. Furthermore, it is speculated that, in addition to the IL-2-induced activation, the BsMAb might provide a necessary co-stimulatory signal by cross-linking the IL-2-activated T lymphocytes to tumour cells through their CD3 complex. In this study we investigated the feasibility of a combination treatment of i.v. BsMAb and s.c. IL-2 therapy. To prevent aspecific immune activation or toxicity that could arise from binding of the BsMAb through its Fc part to FcR-positive cells such as monocytes, F(ab')$_2$ fragments of BIS-1 were used in the present study. Acute toxicity, however, was encountered after administration of BIS-1 F(ab')$_2$ with chills, peripheral vasoconstriction, dyspnoea, fever and release of cytokines such as TNF-α and IFN-γ. These phenomena led to the conclusion that the MTD of BIS1 F(ab')$_2$ given as a 2 h infusion is 5 μg kg$^{-1}$. Recently, other investigators have reported severe toxicity of a similar nature with high systemic release of TNF-α and IFN-γ after administration of 1 mg of a F(ab')$_2$ BsMAb in a patient with ovarian carcinoma. In this study no concomitant IL-2 was given (Tibben *et al.*, 1993). In the present study we also found elevated levels of TNF-α and IFN-γ. It remains to be resolved whether the observed toxicity in the higher doses of the present study is still the result of aspecific stimulation of the monocyte/macrophage system or whether it is induced by specific BIS-1 F(ab')$_2$-mediated T-cell activation, or both. The observed toxicity appears to be unrelated to MOC31 binding alone, since the EGP-2-recognising antibody MOC31 can be given to patients in a dose of at least 50 mg kg$^{-1}$ without any toxic side-effects

(unpublished results). Anti-CD3-directed antibodies (complete Ig with an intact Fc portion), however, have shown to be toxic in cancer patients with a MTD of also approximately 5 μg $kg^{-1}$ (400 μg total dose). However, neurotoxicity with an onset of 12 h after infusion was dose limiting in this setting (Buter *et al.*, 1993). The observed toxicity therefore appears to be related to the combined (i.e. bispecific) binding activity of BIS-1 $F(ab')_2$. T-cell activation, which could possibly induce toxicity, requires not only binding of BIS-1 $F(ab')_2$ to CD3 but also clustering of the CD3 molecules (Schwab *et al.*, 1985). Since the BIS-1 $F(ab')_2$ has only a monovalent binding site to the CD3 molecule, clustering of CD3 molecules and therefore triggering of T-cell activation is thought to be possible only by immobilisation of BIS-1 $F(ab')_2$ via its EGP-2 binding site. Since no EGP-2-positive circulating (blood) cells are present, it is speculated that BIS-1 $F(ab')_2$ might have reacted with tumour cells, or possibly some normal epithelia, after leaving the blood circulation. Subsequently, either *in situ* or newly extravasated T lymphocytes could have been triggered through cross-linking of the CD3 complexes by BIS-1. A more likely explanation is that BIS-1 $F(ab')_2$ bound preferentially to T lymphocytes in the circulation. After extravasation of some of these cells, possibly induced by IL-2, these BIS-1 $F(ab')_2$-loaded cells may react with EGP-2 on tumour or epithelia. The tumour-T cell interaction might subsequently trigger the production of secondary cytokines such as TNF-α and IFN-γ, resulting in the enhanced extravasation of both lymphocytes and monocytes, as we observed in the present study. *In vivo* binding of BIS-1 $F(ab')_2$ to peripheral blood T lymphocytes was evaluated *ex vivo* both functionally by a $^{51}$Cr-release assay and immunocytologically using a sensitive indirect staining method for flow cytometry. For the $^{51}$Cr-release assay, performed to evaluate also functional binding of BIS-1, PBMCs obtained from the patients just prior to and directly after the infusion of BIS-1 were used. Figure 3 shows that *in vivo* BIS-1 $F(ab')_2$-loaded T lymphocytes were able to specifically lyse EGP-2-positive tumour cells *in vitro*. Although the E T ratio of 100:1 is of little clinical relevance, it is indicative for the functional binding of BIS-1 $F(ab')_2$ *in vivo*. At 3 μg $kg^{-1}$ BIS-1 $F(ab')_2$ up to approximately 45% of the maximal obtainable cytotoxicity against EGP-2-positive target cells could be measured. Owing to the partial loss of BIS-1 $F(ab')_2$ during isolation (see Figure 2), this finding is probably an underestimation of the actual *in vivo* capacity to exert anti-tumour activity. The SCLC cell lines GLC-1 and GLC-1M13 are relatively resistant to LAK activity (own observation), and as a result no cytotoxicity was measured in the absence of BIS-1 $F(ab')_2$, which might have been expected because of the *in vivo* activation of NK cells by IL-2. At 5 μg $kg^{-1}$ the target cell killing capacity of PBMCs after the infusion was found to be approximately 50% of the cytotoxicity observed with addition of an optimal concentration of BIS-1 to the assay. However, this was mainly because of the substantially lower maximal inducible cytotoxicity after the infusion with BIS-1 $F(ab')_2$ as compared with the maximal cytotoxic capacity before the infusion at this dose.

This last phenomenon appears to be related to the observed rapid reduction in the number of PBMCs in the peripheral blood during the infusion of 5 μg $kg^{-1}$ BIS-1 $F(ab')_2$. A reduction in PBMCs occurred to a much lesser extent at 3 μg $kg^{-1}$ BIS-1 $F(ab')_2$ (see Figures 5 and 6). However, the low number of circulating PBMCs itself cannot explain the reducing killing capacity of the isolated PBMCs since the cytotoxicity assays were performed at a fixed E T ratio. Lymphocyte subset analysis of blood samples taken before and after infusion with 5 μg $kg^{-1}$ BIS-1 $F(ab')_2$ showed, in addition to the general disappearance of PBMCs, a preferential reduction in the percentage of LFA-1α-bright and HLA-DR-positive cells within the CD3 CD8 double-positive lymphocyte population (see Table III), whereas the percentage of CD3- and CD8-positive cells was not altered by the infusion. The LFA-1α-bright T-cell population has been described as the main population responsible for cytolytic activity (Morimoto *et al.*, 1987; Tsubota *et al.*, 1989). The importance of LFA-1 for the induction of target cell lysis is also indicated from the observation that ICAM-1-negative target cells are relatively resistant to lysis by BsMAb-redirected cytolytic effector cells (Stotter *et al.*, 1989; Braakman *et al.*, 1990; Rivoltini *et al.*, 1991; Webb *et al.*, 1991). Furthermore, LFA-1α-bright cells have been shown to leave the circulation during acute immune responses (Hviid *et al.*, 1991). As discussed above, extravasation of these cells might have been initiated by the TNF-α produced during the infusion with BIS-1. TNF-α is known to induce up-regulation of endothelial adhesion molecules such as ICAM-1 and E-selectin that are involved in the migration of leucocytes (Nickoloff & Griffiths, 1989; Vogetseder *et al.*, 1989; Graeves, 1992; Zimmerman *et al.*, 1992). Since LFA-1α-bright cells have high avidity for both ICAM-1 and -2 (Diamond *et al.*, 1991), this might explain the reduction of especially CD3 CD8 LFA-1α-bright positive T lymphocytes from the circulation, which subsequently explains the observed decreased maximal tagret cell killing *in vitro* after infusion with 5 μg $kg^{-1}$ BIS-1 $F(ab')_2$. The production of IFN-γ in addition to TNF-α, as observed after treatment with 5 μg $kg^{-1}$ BIS-1 $F(ab')_2$, can be taken as a further indication of BIS-1-induced T-cell activation.

We observed one partial response in 14 evaluable patients who were unresponsive to initial s.c. IL-2 monotherapy. This is in line with the cytotoxic capacity of the redirected cells observed *in vitro*, although a late response to IL-2 cannot be excluded. BsMAb-redirected T lymphocytes, in contrast to genuine HLA-restricted cytotoxic T lymphocytes, are able to exert their lytic capacity only once towards a relevant target cell. Redirected killing capacity can be restored, however, by adding new BsMAbs (Blank-Voorthuis *et al.*, 1993). Therefore, to obtain better anti-tumour responses, it might be necessary to give BIS-1 $F(ab')_2$ more than once to the patient.

In conclusion, BIS-1 $F(ab')_2$ can be given to cancer patients with a MTD of 5 μg $kg^{-1}$ as a 2 h infusion, when combined with IL-2. BIS-1 $F(ab')_2$ can be detected on peripheral blood T lymphocytes both cytologically and functionally upon i.v. administration of the BsMAb. At 5 μg $kg^{-1}$ BIS-1 $F(ab')_2$ a rapid induction of TNF-α and IFN-γ production is observed and a transient leucopenia with a preferential depletion of LFA-1α-bright CD8-positive T lymphocytes from the blood is seen. The wide applicability of the EGP-2 antibody warrants further investigations to fully exploit the full potential of this kind of immunotherapy.

The authors acknowledge G. Mesander for excellent technical assistance with flow cytometric analysis and J. Kosterink for advice concerning pharmaceutical aspects of BIS-1 $F(ab')_2$. Large-scale production of BIS-1 and isolation of the bispecific antibody fraction was done in cooperation with Dr R. vd Griend (Biocult, Utrecht) and Dr W. de Vrij and Drs M.W.A. de Jonge (MCA development, Groningen). This study was supported by a grant from the Dutch Cancer Society (Koningin Wilhelmina Fonds, 89-07).

### References

ANONYMOUS (1979). *WHO Handbook for Reporting Results of Cancer Treatment*. Martinus Nijhoff: The Hague.

BACH, F.H., GELLER, R.L., NELSON, P.J., PANZER, G.G., BENFIELD, M.R., INVERARDI, L., PODACK, E.R., WITSON, J.C., HOUCHINS, J.P. & ALTER, B.J. (1989). A minimal stepwise activation analysis of functional maturation of T lymphocytes. *Immunol. Rev.*, **111**, 35-57.

BEVERLY, P.C.L., SOUHAMI, R.L. & BOBROW, L. (1988). Proceedings of the first international workshop on Small Cell Lung Cancer Antigens. In *Lung Cancer*, Souhami, R.L., Beverley, L. & Bobrow, L. (eds) pp. 15-36. Elseviers Science Publishers: Amsterdam.

BLANK-VOORTHUIS, C.J.A.C., BRAAKMAN, E., RONTELTAP, C.P.M., TILLY, B.C., STURM, E., WARNAAR, S.O. & BOLHUIS, R.L.H. (1993). Clustered CD3/TCR complexes do not transduce activation signals following bispecific monoclonal antibody triggered lysis by CTL via CD3. *J. Immunol.*, **151 (6)**, 2904-2914.

BOLHUIS, R.L.H., STURM, E. & BRAAKMAN, E. (1991). T cell targeting in cancer therapy. *Cancer Immunol. Immunother.*, **34**, 1-8.

BOLHUIS, R.L.H., LAMERS, C.H.L., GOEY, S.H., EGGERMONT, A.M.M., TRIMBOS, J.B.M.Z., STOTER, G., LANZEVECCHIA, A., DI RE, E., MIOTTI, S., RASPAGLIESI, F., RIVOLTINI, L. & COLNAGHI, M.I. (1992). Adoptive immunotherapy of ovarian carcinoma with bs-Mab-targeted lymphocytes: a multicentre study. *Int. J. Cancer*, (Suppl. 7), 78-81.

BRAAKMAN, E., GOEDEBUURE, P.S., VREUGDEHIL, R.J., SEGAL, D.M. & BOLHUIS, R.L.H. (1990). ICAM melanoma cells are relatively resistant to CD3-mediated T-cell lysis. *Int. J. Cancer*, **46**, 475-480.

BUTER, J., JANSSEN, R.A.J., MARTENS, A., SLEIJFER, D.T., DE LEIJ, L. & MULDER, N.H. (1993). Phase I/II of low dose intravenous OKT3 and subcutaneous interleukin-2 in metastatic cancer. *Eur. J. Cancer*, **29A**, 2108-2113.

DE JONG, R., BROUWER, M., REBEL, V.I., VAN SEVENTER, G.A., MIEDEMA, F. & VAN LIER, R.A.W. (1990). Generation of alloreactive cytolytic T lymphocytes by immobilized anti-CD3 monoclonal antibodies. Analysis of requirements for human T-lymphocyte differentiation. *Immunology*, **70**, 357-364.

DE LAU, W.J.M., VAN LOON, A.E., HEIJE, K., VALERIO, D. & BAST, B.J. (1989). Production of hybrid hybridomas on HAT$^s$-neomycin$^r$ double mutants. *J. Immunol.*, **117**, 1-8.

DE LEIJ, L., POSTMUS, E.P., BUYS, C.H.C.M., ELEMA, J.D., RAMAEKERS, F., POPPEMA, S., BROUWER, M., VAN DER VEEN, A.Y., MESANDER, G. & THE, T.H. (1985). Characterization of three new variant cell lines derived from small cell carcinoma of the lung. *Cancer Res.*, **45**, 6024-6033.

DE LEIJ, L., HELFRICH, W., STEIN, R. & MATTES, M.J. (1993). SCLC cluster 2 antibodies detect the pancarcinoma/epithelial glycoprotein EGP-2. *Int. J. Cancer* (in press).

DIAMOND, M.S., STAUNTON, D.E., MARLIN, S.D. & SPRINGER, T.A. (1991). Binding of the integrin Mac-1 (CD11b/CD18) to the third immunoglobulin-like domain of ICAM-1 (CD54) and its regulation by glycosylation. *Cell*, **65**, 961-971.

FERRINI, S., PRIGIONE, I., MIOTTI, S., CICCONE, E., CANTONI, C., CHEN, Q., COLNAGHI, M.I. & MORETTA, L. (1991). Bispecific monoclonal antibodies directed to CD16 and to a tumor-associated antigen induce target-cell lysis by resting NK cells and by a subset of NK clones. *Int. J. Cancer*, **48**, 227-233.

FISHER, B., PACKARD, B.S., CARRASQUILLO, J.A., CARTER, C.S., TOPALIAN, S.L., YANG, J.C., YOLLES, P., LARSON, S.M. & ROSENBERG, S.A. (1989). Tumor localization of adoptively transferred indium-111 labeled tumor infiltrating lymphocytes in patients with metastatic melanoma. *J. Clin. Oncol.*, **7**, 250-261.

GRAEVES, M.W. (1992). Early cellular and molecular events in inflamed skin: an integrated concept. *J. Dermatol. Sci.*, **3**, 1-5.

HERLYN, M., STEPLEWSKI, Z., HERLYN, D. & KOPROWSKI, H. (1979). Colorectal carcinoma specific antigen: detection by means of monoclonal antibodies. *Proc. Natl Acad. Sci. USA*, **75**, 1438-1452.

HVIID, L., THEANDER, T.G., ABDULHADI, N.H., ABU-ZEID, Y.A., BAYOUMI, R.A. & JENSEN, J.B. (1991). Transient depletion of T cells with high LFA-1 expression from peripheral circulating during acute plasmodium falciparum malaria. *Eur. J. Immunol.*, **21**, 1249-1253.

JANSSEN, R.A.J., BUTER, J., STRAATSMA, E., HEIJN, A.A., SLEIJFER, D. TH., DE VRIES, E.G.E., MULDER, N.H., THE, T.H. & DE LEIJ, L. (1993). HLA-DR-expressing CD8 bright cells are only temporarily present in the circulation during subcutaneous interleukin-2 therapy in renal cell carcinoma patients. *Cancer Immuno. Immunother.*, **36**, 198-204.

KERR, L., HUNTOON, C., DONOHUE, J., LEIBSON, P.J., BANDER, N.H., GHOSE, T., LUNER, S.J., VESSELLA, R. & MCKEAN, D.J. (1990). Heteroconjugate antibody-directed killing of autologous human renal carcinoma cells by *in vitro*-activated lymphocytes. *J. Immunol.*, **144**, 4060-4067.

KROESEN, B.J., TER HAAR, A., SPAKMAN, H., WILLEMSE, P.H.B., SLEIJFER, D. TH., DE VRIES, E.G.E. & MULDER, N.H. (1993). Local antitumour treatment in carcinoma patients with bispecific monoclonal antibody redirected T-cells. *Cancer Immunol. Immunother.*, **37**, 400-407.

MEZZANZANICA, D., GARRIDO, M.A., NEBLOCK, D.S., DADDONA, P.E., ANDREW, S.M., ZURAWSKI, C.R., SEGAL, D.M. & WUNDERLICH, J.R. (1991). Human T-Lymphocytes targeted against an established human ovarian carcinoma with a bispecific F(ab')2 antibody prolong host survival in a murine xenograft model. *Cancer Res.*, **51(20)**, 5716-5721.

MORIMOTO, C., RUDD, C.E., LETVIN, N.L. & SCHLOSSMANN, S.F. (1987). A novel epitope of the LFA-1 antigen which can distinguish killer effector and suppressor cells in human CD8 cells. *Nature*, **330**, 479-482.

NICKOLOFF, B.J. & GRIFFITHS, C.E.M. (1989). T lymphocytes and monocytes bind to keratinocytes in frozen sections of biopsy specimens in normal and inflamed skin: modulation by recombinant gamma interferon and tumour necrosis factor. *J. Am. Acad. Dermatol.*, **20**, 617-629.

NITTA, T., SATO, K., YAGITA, H., OKUMURA, K. & ISHII, S. (1990). Preliminary trial of specific targeting therapy against malignant glioma. *Lancet*, **335**, 368-371.

PANKONIN, G., REIPERT, B. & AGAR, A. (1990). Interaction between interleukin-2 activated lymphocytes and vascular endothelium: binding to and migration across specialized and non-specialized endothelia. *Immunology*, 7751-7760.

RIVOLTINI, L., CATTORETTI, F.A., MASTROIOANNI, A., MELANI, C., COLOMBO, M.P. & PARMIANI, G. (1991). The high lysability by LAK cells of colon-carcinoma cells resistant to doxorubicin is associated with a high expression of ICAM-1, LFA-3, NCA and to a less-differentiated phenotype. *Int. J. Cancer*, **47**, 746-754.

ROSENBERG, S.A., LOTZE, M.T., YANG, J.C., AEBERSOLD, P.M., LINEHAN, W.M., SEIPP, C.A. & WHITE, D.E. (1989). Experience with the use of high-dose interleukin-2 in the treatment of 652 cancer patients. *Ann. Surg.*, **210**, 474-485.

SAMONIGG, H., WILDERS-TRUSSCHING, M., LOIBNER, H., PLOT, R., ROT, A., KUSS, I., WERNER, G., STOGER, H., WRANN, M. & HERLYN, D. (1992). Immune response to tumor antigens in a patient with colorectal cancer after immunization with anti-idiotype antibody. *Clin. Immunol. Immunopathol.*, **65**, 271-277.

SCHWAB, R., CROW, M.K., RUSSO, C. & WEKSLER, M.E. (1985). Requirements for T cell activation by OKT3 monoclonal antibody: role of modulation of T3 molecules and interleukin 1. *J. Immunol.*, **135**, 1714-1718.

SEGAL, D.M., QIAN, J.H., ANDREW, S.M., TITUS, J.A., MEZZANZANICA, D., GARRIDO, M.A. & WUNDERLICH, J.R. (1991). Cytokine release by peripheral blood lymphocytes targeted with bispecific antibodies, and its role in blocking tumor growth. *Ann. NY Acad. Sci.*, **636**, 288-291.

SINDELAR, W.F., MAHER, M.M., HERLYN, D., SEARS, H.F., STEPLEWSKI, Z. & KOPROWSKI, H. (1986). Trial of therapy with monoclonal antibody 17-1A in pancreatic carcinoma: preliminary results. *Hybridoma*, (Suppl.) **1p**, 125-132.

SLEIJFER, D.T., JANSSEN, R.A., BUTER, J., DE VRIES, E.G.E., WILLEMSE, P.H.B. & MULDER, N.H. (1992). Phase II study of subcutaneous interleukin-2 in unselected patients with advanced renal cell cancer on an outpatient basis. *J. Clin. Oncol.*, **10**, 1119-1123.

STEPLEWSKI, Z., CHANG, T.H., HERLYN, M. & KOPROWSKI, H. (1981). Release of antibody-defined antigens by human colorectal carcinoma and melanoma cells. *Cancer Res.*, **41**, 2723-2727.

STOTTER, H., WIEBKE, E.A., TOMITA, S., BELLDEGRUN, A., TOPALIAN, S., ROSENBERG, S.A. & LOTZE, M.T. (1989). Cytokines alter target cell susceptibility to lysis. Evaluation of tumor infiltrating lymphocytes. *J. Immunol.*, **142**, 1767-1773.

TAX, W.J.M., WILLEMS, H.W., REEKERS, P.P.M., CAPEL, P.J.A. & KOENE, R.H. (1983). Polymorphism and mitogenic effect of IgG monoclonal antibodies against T3 antigen on human T-cells. *Nature*, **304**, 445-447.

TIBBEN, J.G., BOERMAN, O.C., CLAESSENS, R.A.M.J., CORSTENS, F.H.M., VAN DEUREN, M., DE MULDER, P.H.M., VAN DER MEER, J.W.M., KEIJSER, K.G.G. & MASSUGER, L.F.A.G. (1993). Cytokine release in an ovarian carcinoma patient following intravenous administration of bispecific antibody OC/TR F(ab')$_2$. *J. Natl Cancer Inst.*, **85**, 1003-1004.

TSUBOTA, H., LORD, C.I., WATKINS, C.M., MORIMOTO, C. & LETVIN, L. (1989). A cytotoxic T-lymphocyte inhibits acquired immunodeficiency syndrome virus replication in peripheral blood lymphocytes. *J. Exp. Med.*, **169**, 1421-1434.

VARKI, N.M., REISVELD, R.A. & WALKER, L.E. (1984). Antigens associated with a human lung adenocarcinoma defined by monoclonal antibodies. *Cancer Res.*, **44**, 681-687.

VOGETSEDER, W., FEICHTINGER, H., SCHULZ, T.F., SCHWAEBLE, W., TABACZEWSKI, P., MITTERER, M., BOCK, G., MARTH, CH., DAPUNT, O., MIKUZ, G. & DIERICH, M.P. (1989). Expression of 7F7-antigen, a human adhesion molecule identical to intracellular adhesion molecule-1 (ICAM-1), in human carcinomas and their stromal fibroblasts. *Int. J. Cancer*, **43**, 768–773.

WEBB, D.S.A., MOTOWSKI, H.S. & GERRARD, TH. L. (1991). Cytokine-induced enhancements of ICAM-1 expression results in increased vulnerability of tumor cells to monocyte mediated lysis. *J. Immunol.*, **146**, 3682–3686.

WEINER, G.J. & HILLSTROM, J.R. (1991). Bispecific anti-idiotype/anti-CD3 antibody therapy of murine B cell lymphoma. *J. Immunol.*, **147**, 4035–4044.

WEINER, L.M., HOLMES, M., ADAMS, G.P., LACRETA, F.L., WATTS, P. & PALAZZO, I.G. (1993). A human tumor xenograft model of therapy with a bispecific monoclonal antibody targeting c-erb-2 and CD16. *Cancer Res.*, **52**, 94–100.

ZIMMERMAN, G.A., PRESCOT, S.A. & MCINTYRE, T.M. (1992). Endothelial cell interactions with granulocytes. Tethering and signaling molecules. *Immunol. Today*, **13**, 93–100.

ZOLA, H., NEOH, S.H., MANZIORIS, B.X., WEBSTER, J. & LOUGHAN, M.S. (1990). Detection by immunofluorescence of surface molecules present in low copy numbers. High sensitivity staining and calibration of flow cytometer. *J. Immunol. Methods*, **135**, 247–255.


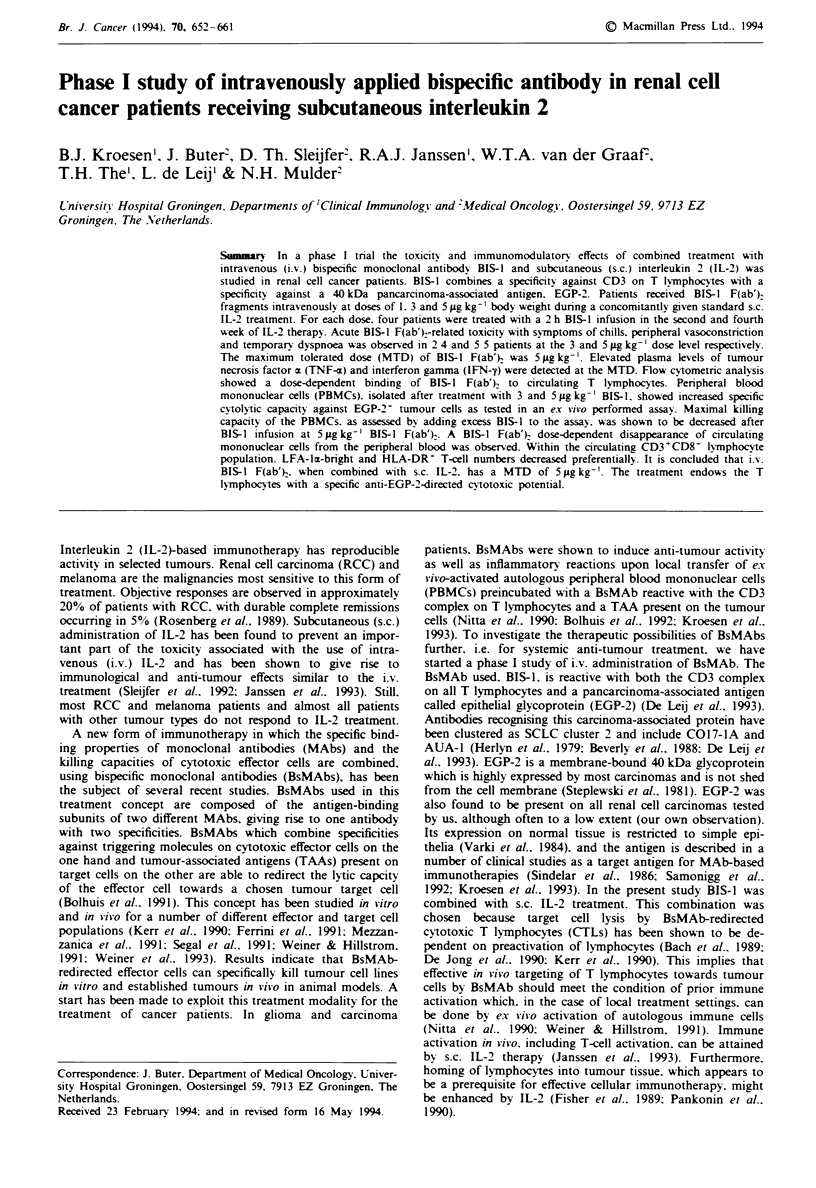

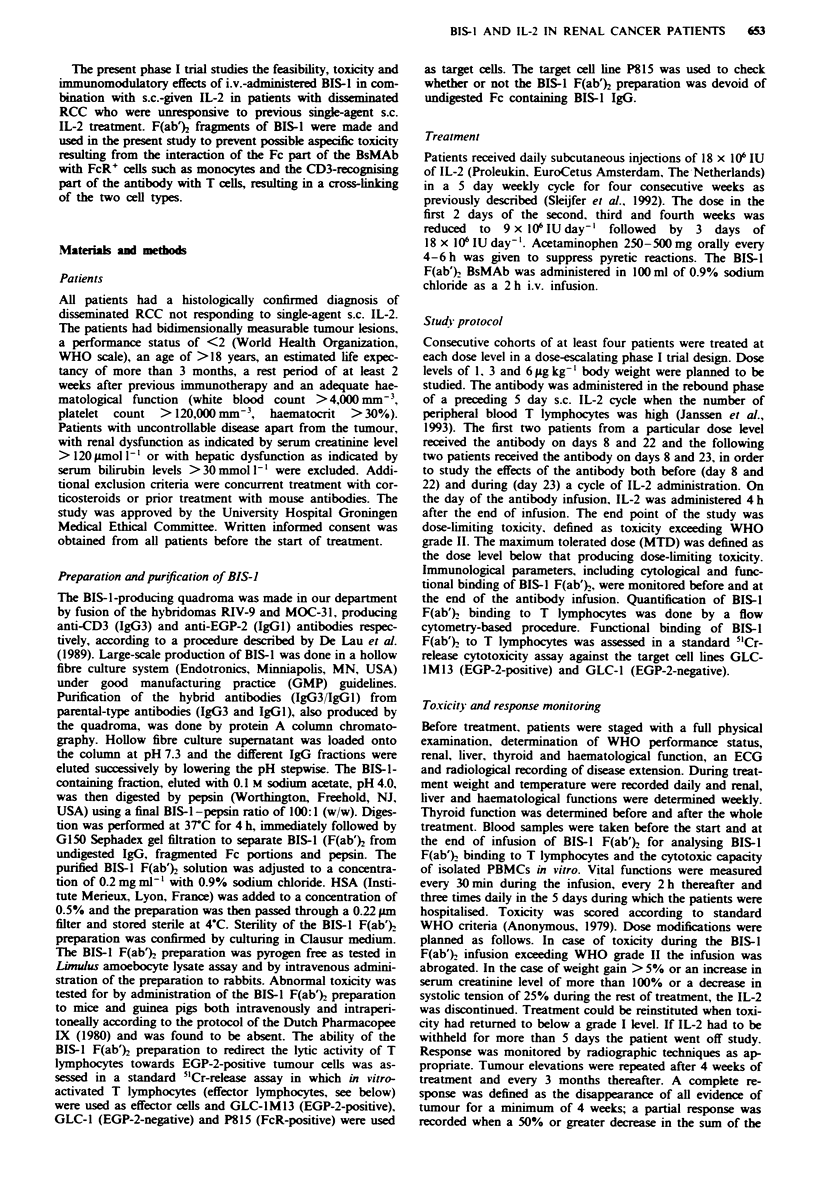

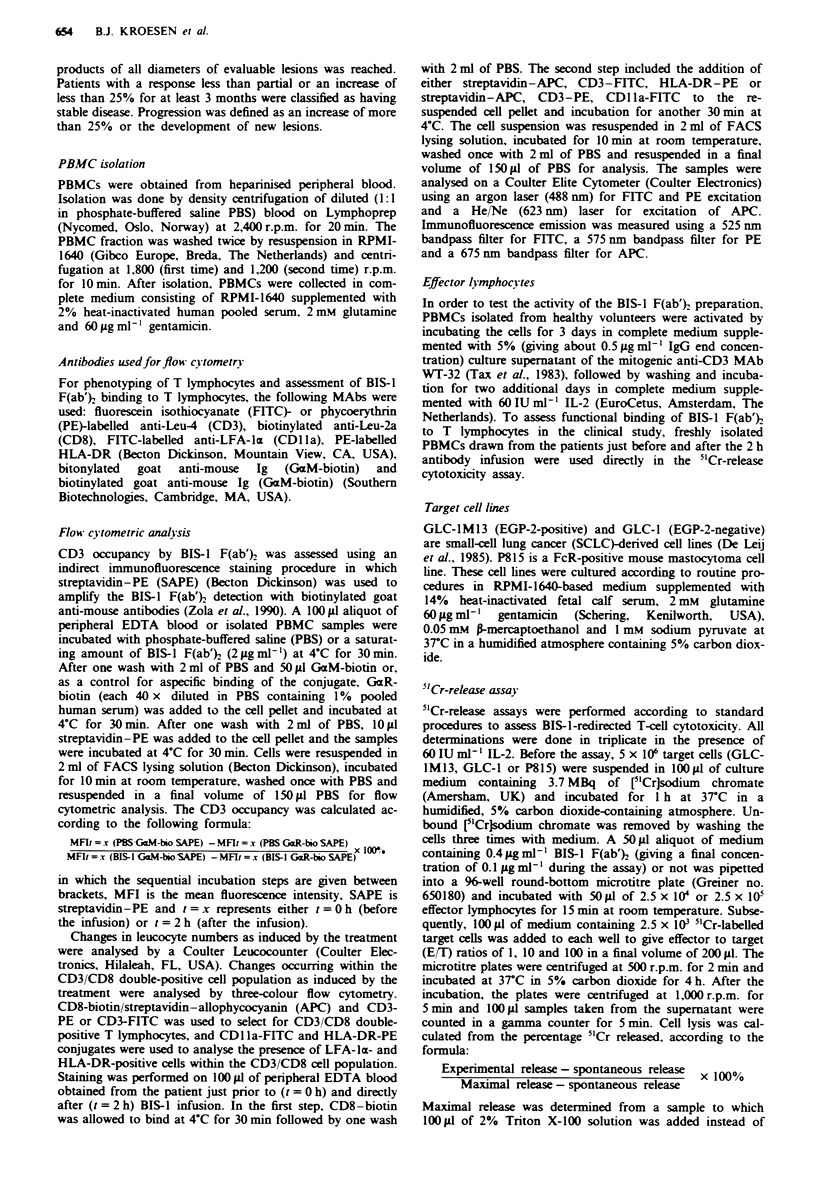

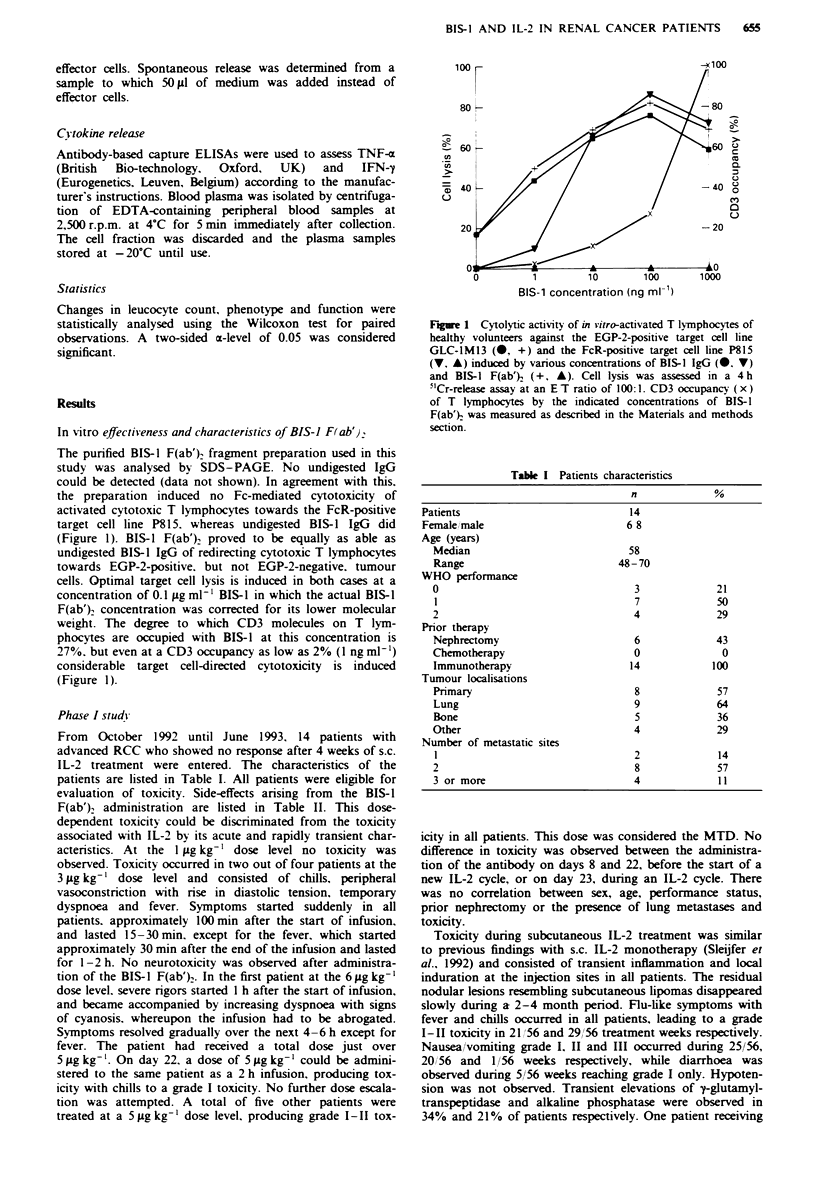

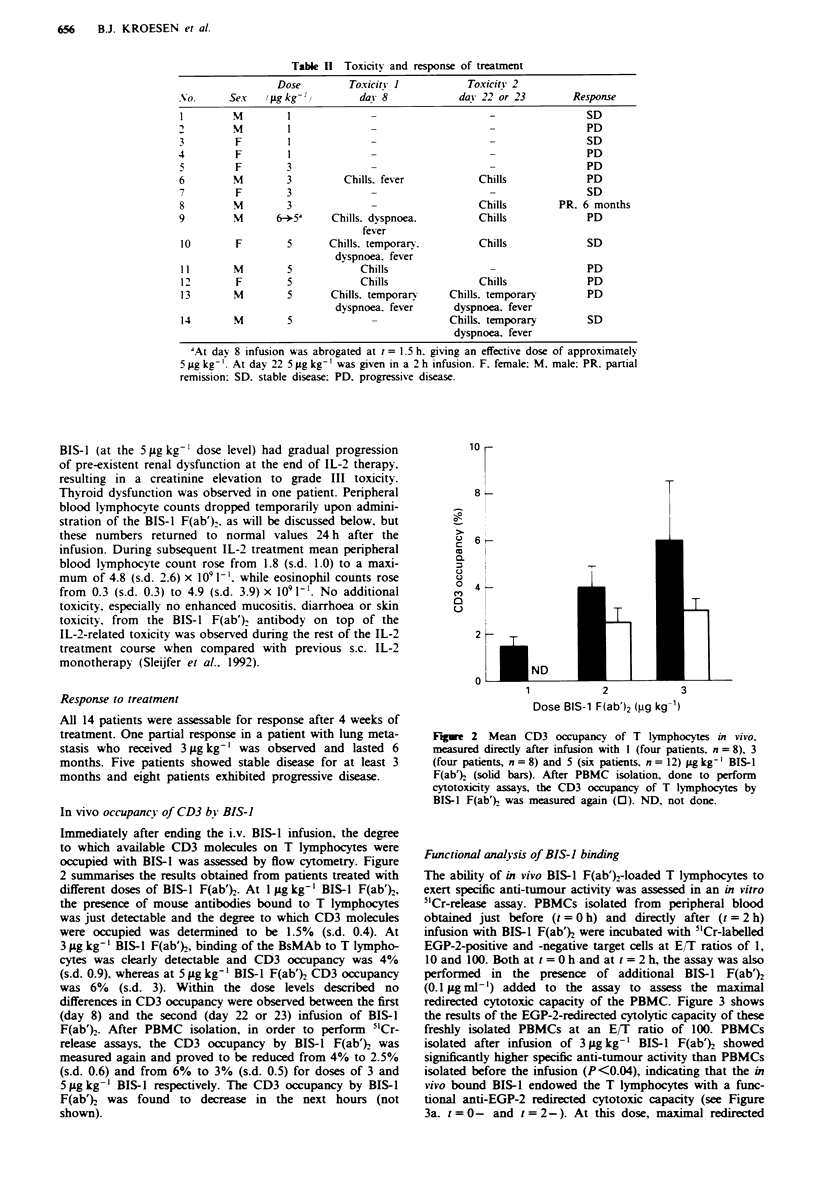

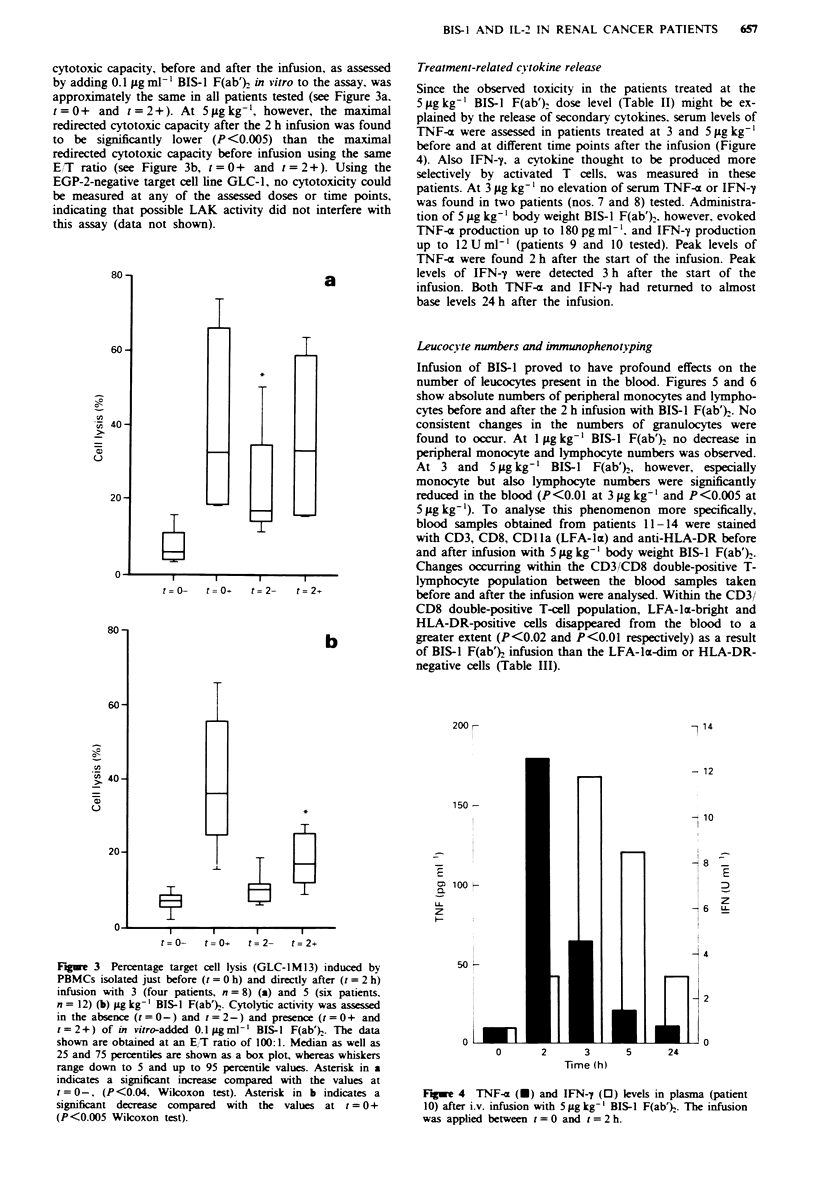

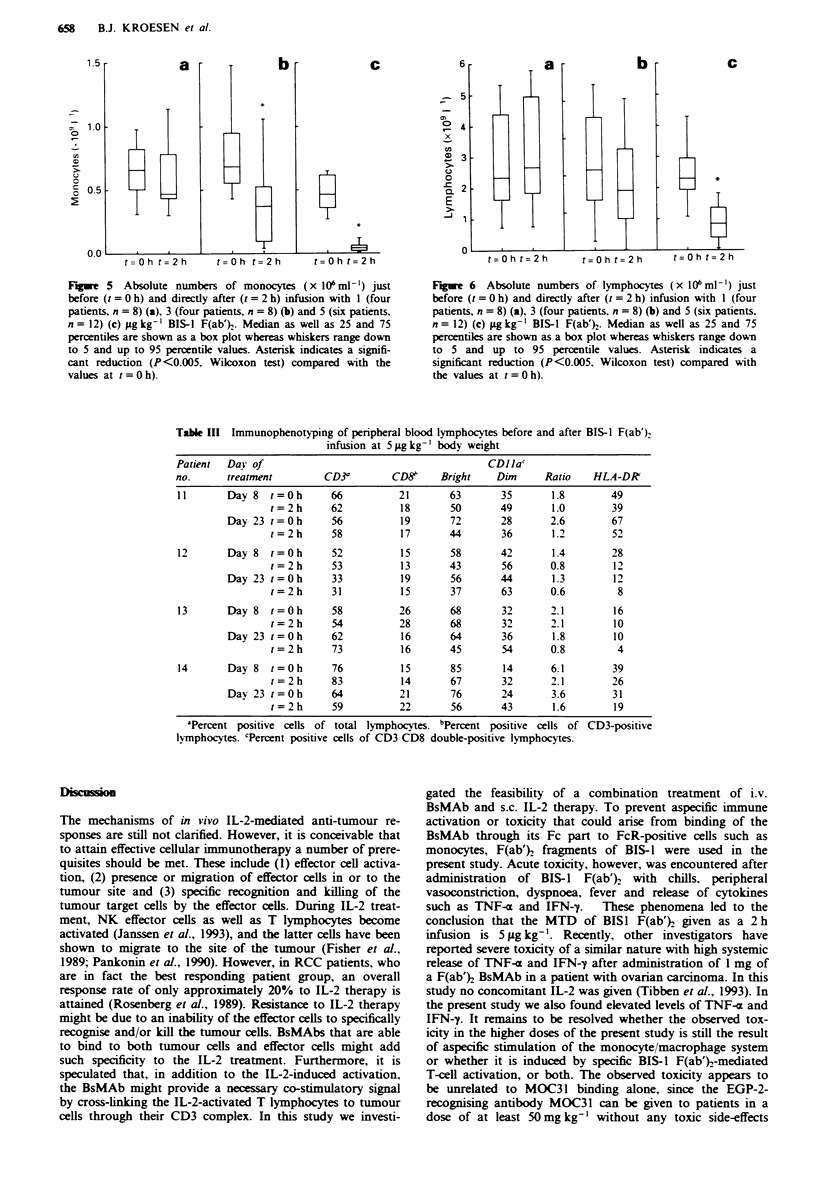

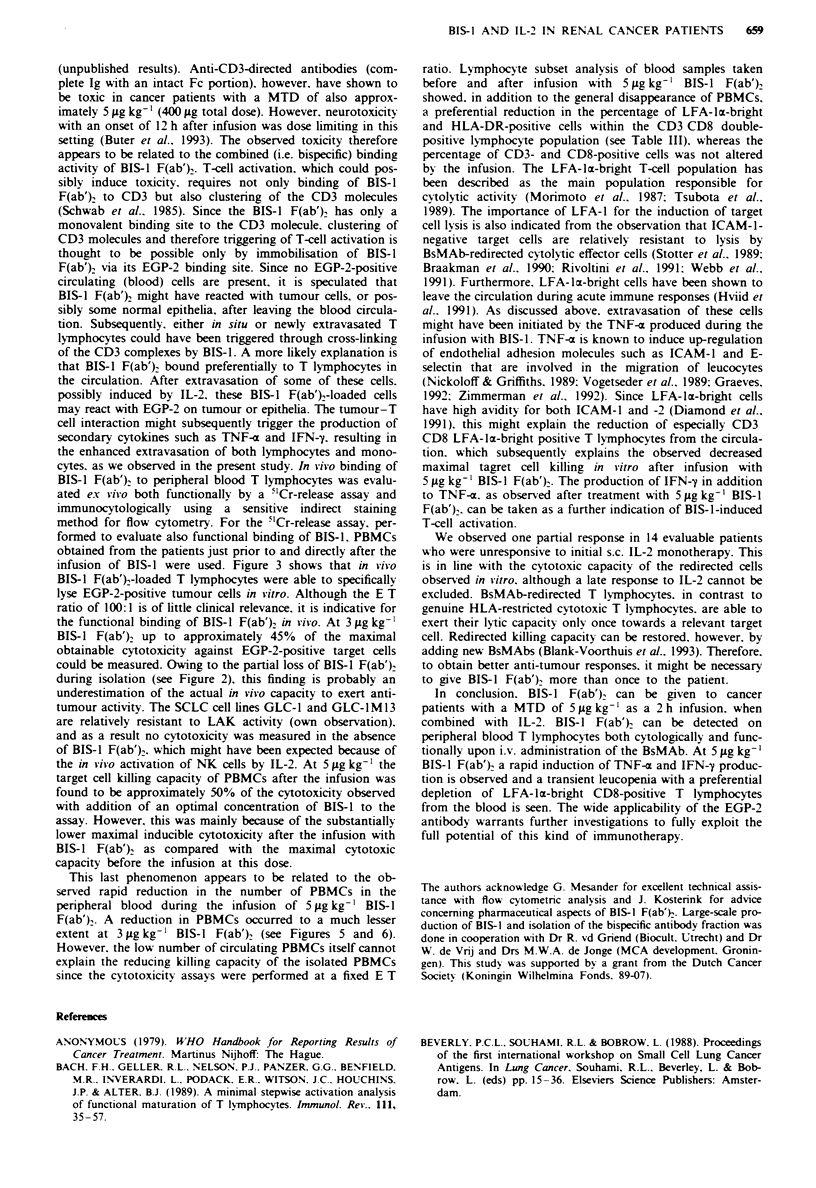

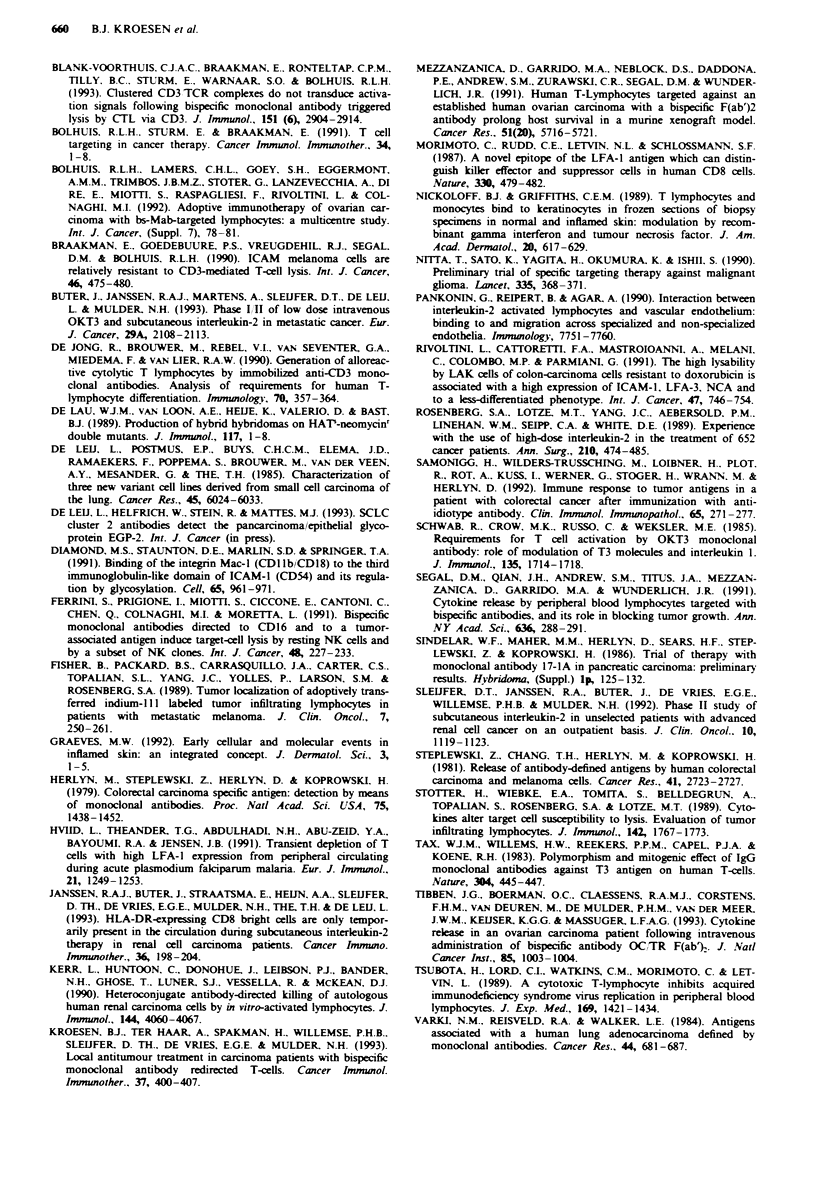

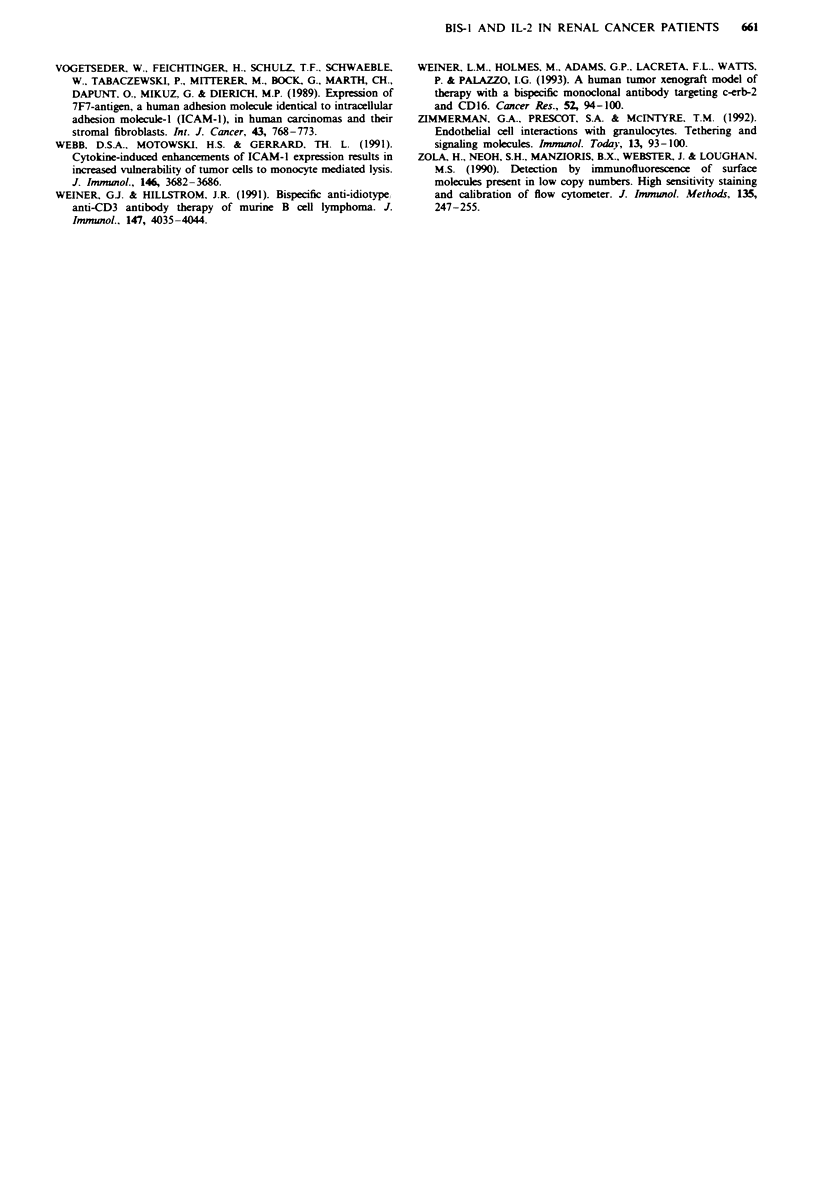


## References

[OCR_01197] Bach F. H., Geller R. L., Nelson P. J., Panzer S., Gromo G., Benfield M. R., Inverardi L., Podack E. R., Witson J. C., Houchins J. P. (1989). A "minimal signal-stepwise activation" analysis of functional maturation of T lymphocytes.. Immunol Rev.

[OCR_01215] Blank-Voorthuis C. J., Braakman E., Ronteltap C. P., Tilly B. C., Sturm E., Warnaar S. O., Bolhuis R. L. (1993). Clustered CD3/TCR complexes do not transduce activation signals after bispecific monoclonal antibody-triggered lysis by cytotoxic T lymphocytes via CD3.. J Immunol.

[OCR_01225] Bolhuis R. L., Lamers C. H., Goey S. H., Eggermont A. M., Trimbos J. B., Stoter G., Lanzavecchia A., di Re E., Miotti S., Raspagliesi F. (1992). Adoptive immunotherapy of ovarian carcinoma with bs-MAb-targeted lymphocytes: a multicenter study.. Int J Cancer Suppl.

[OCR_01220] Bolhuis R. L., Sturm E., Braakman E. (1991). T cell targeting in cancer therapy.. Cancer Immunol Immunother.

[OCR_01236] Braakman E., Goedegebuure P. S., Vreugdenhil R. J., Segal D. M., Shaw S., Bolhuis R. L. (1990). ICAM- melanoma cells are relatively resistant to CD3-mediated T-cell lysis.. Int J Cancer.

[OCR_01241] Buter J., Janssen R. A., Martens A., Sleijfer D. T., de Leij L., Mulder N. H. (1993). Phase I/II study of low-dose intravenous OKT3 and subcutaneous interleukin-2 in metastatic cancer.. Eur J Cancer.

[OCR_01248] De Jong R., Brouwer M., Rebel V. I., Van Seventer G. A., Miedema F., Van Lier R. A. (1990). Generation of alloreactive cytolytic T lymphocytes by immobilized anti-CD3 monoclonal antibodies. Analysis of requirements for human cytolytic T-lymphocyte differentiation.. Immunology.

[OCR_01269] Diamond M. S., Staunton D. E., Marlin S. D., Springer T. A. (1991). Binding of the integrin Mac-1 (CD11b/CD18) to the third immunoglobulin-like domain of ICAM-1 (CD54) and its regulation by glycosylation.. Cell.

[OCR_01275] Ferrini S., Prigione I., Miotti S., Ciccone E., Cantoni C., Chen Q., Colnaghi M. I., Moretta L. (1991). Bispecific monoclonal antibodies directed to CD16 and to a tumor-associated antigen induce target-cell lysis by resting NK cells and by a subset of NK clones.. Int J Cancer.

[OCR_01285] Fisher B., Packard B. S., Read E. J., Carrasquillo J. A., Carter C. S., Topalian S. L., Yang J. C., Yolles P., Larson S. M., Rosenberg S. A. (1989). Tumor localization of adoptively transferred indium-111 labeled tumor infiltrating lymphocytes in patients with metastatic melanoma.. J Clin Oncol.

[OCR_01290] Greaves M. W. (1992). Early cellular and molecular events in inflamed skin: an integrated concept.. J Dermatol Sci.

[OCR_01297] Herlyn M., Steplewski Z., Herlyn D., Koprowski H. (1979). Colorectal carcinoma-specific antigen: detection by means of monoclonal antibodies.. Proc Natl Acad Sci U S A.

[OCR_01304] Hviid L., Theander T. G., Abdulhadi N. H., Abu-Zeid Y. A., Bayoumi R. A., Jensen J. B. (1991). Transient depletion of T cells with high LFA-1 expression from peripheral circulation during acute Plasmodium falciparum malaria.. Eur J Immunol.

[OCR_01311] Janssen R. A., Buter J., Straatsma E., Heijn A. A., Sleijfer D. T., de Vries E. G., Mulder N. H., The T. H., de Leij L. (1993). HLA-Dr-expressing CD8bright cells are only temporarily present in the circulation during subcutaneous recombinant interleukin-2 therapy in renal cell carcinoma patients.. Cancer Immunol Immunother.

[OCR_01316] Kerr L., Huntoon C., Donohue J., Leibson P. J., Bander N. H., Ghose T., Luner S. J., Vessella R., McKean D. J. (1990). Heteroconjugate antibody-directed killing of autologous human renal carcinoma cells by in vitro-activated lymphocytes.. J Immunol.

[OCR_01323] Kroesen B. J., ter Haar A., Spakman H., Willemse P., Sleijfer D. T., de Vries E. G., Mulder N. H., Berendsen H. H., Limburg P. C., The T. H. (1993). Local antitumour treatment in carcinoma patients with bispecific-monoclonal-antibody-redirected T cells.. Cancer Immunol Immunother.

[OCR_01333] Mezzanzanica D., Garrido M. A., Neblock D. S., Daddona P. E., Andrew S. M., Zurawski V. R., Segal D. M., Wunderlich J. R. (1991). Human T-lymphocytes targeted against an established human ovarian carcinoma with a bispecific F(ab')2 antibody prolong host survival in a murine xenograft model.. Cancer Res.

[OCR_01338] Morimoto C., Rudd C. E., Letvin N. L., Schlossman S. F. (1987). A novel epitope of the LFA-1 antigen which can distinguish killer effector and suppressor cells in human CD8 cells.. Nature.

[OCR_01351] Nitta T., Sato K., Yagita H., Okumura K., Ishii S. (1990). Preliminary trial of specific targeting therapy against malignant glioma.. Lancet.

[OCR_01365] Rivoltini L., Cattoretti G., Arienti F., Mastroianni A., Melani C., Colombo M. P., Parmiani G. (1991). The high lysability by LAK cells of colon-carcinoma cells resistant to doxorubicin is associated with a high expression of ICAM-1, LFA-3, NCA and a less-differentiated phenotype.. Int J Cancer.

[OCR_01370] Rosenberg S. A., Lotze M. T., Yang J. C., Aebersold P. M., Linehan W. M., Seipp C. A., White D. E. (1989). Experience with the use of high-dose interleukin-2 in the treatment of 652 cancer patients.. Ann Surg.

[OCR_01377] Samonigg H., Wilders-Truschnig M., Loibner H., Plot R., Rot A., Kuss I., Werner G., Stöger H., Wrann M., Herlyn D. (1992). Immune response to tumor antigens in a patient with colorectal cancer after immunization with anti-idiotype antibody.. Clin Immunol Immunopathol.

[OCR_01382] Schwab R., Crow M. K., Russo C., Weksler M. E. (1985). Requirements for T cell activation by OKT3 monoclonal antibody: role of modulation of T3 molecules and interleukin 1.. J Immunol.

[OCR_01389] Segal D. M., Qian J. H., Andrew S. M., Titus J. A., Mezzanzanica D., Garrido M. A., Wunderlich J. R. (1991). Cytokine release by peripheral blood lymphocytes targeted with bispecific antibodies, and its role in blocking tumor growth.. Ann N Y Acad Sci.

[OCR_01399] Sleijfer D. T., Janssen R. A., Buter J., de Vries E. G., Willemse P. H., Mulder N. H. (1992). Phase II study of subcutaneous interleukin-2 in unselected patients with advanced renal cell cancer on an outpatient basis.. J Clin Oncol.

[OCR_01406] Steplewski Z., Chang T. H., Herlyn M., Koprowski H. (1981). Release of monoclonal antibody-defined antigens by human colorectal carcinoma and melanoma cells.. Cancer Res.

[OCR_01411] Stötter H., Wiebke E. A., Tomita S., Belldegrun A., Topalian S., Rosenberg S. A., Lotze M. T. (1989). Cytokines alter target cell susceptibility to lysis. II. Evaluation of tumor infiltrating lymphocytes.. J Immunol.

[OCR_01419] Tax W. J., Willems H. W., Reekers P. P., Capel P. J., Koene R. A. (1983). Polymorphism in mitogenic effect of IgG1 monoclonal antibodies against T3 antigen on human T cells.. Nature.

[OCR_01423] Tibben J. G., Boerman O. C., Claessens R. A., Corstens F. H., van Deuren M., de Mulder P. H., van der Meer J. W., Keijser K. G., Massuger L. F. (1993). Cytokine release in an ovarian carcinoma patient following intravenous administration of bispecific antibody OC/TR F(ab')2.. J Natl Cancer Inst.

[OCR_01433] Tsubota H., Lord C. I., Watkins D. I., Morimoto C., Letvin N. L. (1989). A cytotoxic T lymphocyte inhibits acquired immunodeficiency syndrome virus replication in peripheral blood lymphocytes.. J Exp Med.

[OCR_01437] Varki N. M., Reisfeld R. A., Walker L. E. (1984). Antigens associated with a human lung adenocarcinoma defined by monoclonal antibodies.. Cancer Res.

[OCR_01447] Vogetseder W., Feichtinger H., Schulz T. F., Schwaeble W., Tabaczewski P., Mitterer M., Böck G., Marth C., Dapunt O., Mikuz G. (1989). Expression of 7F7-antigen, a human adhesion molecule identical to intercellular adhesion molecule-1 (ICAM-1) in human carcinomas and their stromal fibroblasts.. Int J Cancer.

[OCR_01452] Webb D. S., Mostowski H. S., Gerrard T. L. (1991). Cytokine-induced enhancement of ICAM-1 expression results in increased vulnerability of tumor cells to monocyte-mediated lysis.. J Immunol.

[OCR_01458] Weiner G. J., Hillstrom J. R. (1991). Bispecific anti-idiotype/anti-CD3 antibody therapy of murine B cell lymphoma.. J Immunol.

[OCR_01463] Weiner L. M., Holmes M., Adams G. P., LaCreta F., Watts P., Garcia de Palazzo I. (1993). A human tumor xenograft model of therapy with a bispecific monoclonal antibody targeting c-erbB-2 and CD16.. Cancer Res.

[OCR_01469] Zimmerman G. A., Prescott S. M., McIntyre T. M. (1992). Endothelial cell interactions with granulocytes: tethering and signaling molecules.. Immunol Today.

[OCR_01474] Zola H., Neoh S. H., Mantzioris B. X., Webster J., Loughnan M. S. (1990). Detection by immunofluorescence of surface molecules present in low copy numbers. High sensitivity staining and calibration of flow cytometer.. J Immunol Methods.

[OCR_01259] de Leij L., Postmus P. E., Buys C. H., Elema J. D., Ramaekers F., Poppema S., Brouwer M., van der Veen A. Y., Mesander G., The T. H. (1985). Characterization of three new variant type cell lines derived from small cell carcinoma of the lung.. Cancer Res.

